# Territorial and Farm-Level Inequalities in Cattle Vaccination and Disease-Control Practices in Ecuador: Evidence from the 2025 National Agricultural Survey

**DOI:** 10.3390/vetsci13080819

**Published:** 2026-08-17

**Authors:** Nestor Acosta, Teresa Guarda

**Affiliations:** Rectorate, Faculty of Systems and Telecommunications, Universidad Estatal Península de Santa Elena, Santa Elena 240204, Ecuador; nacosta@upse.edu.ec

**Keywords:** cattle health, foot-and-mouth disease, cattle vaccination, disease-control practices, veterinary surveillance, complex survey analysis, Manabí, Ecuador

## Abstract

National evidence on cattle vaccination, disease-control practices, and producer-reported disease remains limited in Ecuador. This study assessed holding-level participation in preventive cattle-health activities and factors associated with self-reported disease, focusing on Manabí. We analysed anonymised public-use microdata from the 2025 Continuous Agricultural Production and Area Survey (ESPAC); all four outcomes were based on producer reports and were not independently verified against veterinary or administrative records. The principal design-adjusted analysis included 10,588 positive-weight holdings across continental Ecuador. Survey-weighted proportions and multivariable logistic regression models incorporated expansion weights, strata, and primary sampling units. Estimated participation in the official foot-and-mouth disease vaccination campaign, other cattle vaccination, and disease-control activities was 86.38%, 55.96%, and 64.74%, respectively; 11.22% of holdings reported a principal cattle disease. Larger herd size was positively associated with all four outcomes (all *p* < 0.001). Compared with the Coast, holdings in the Amazon and Highlands had lower adjusted odds of other vaccination and disease-control activities and higher odds of self-reported disease (all *p* ≤ 0.002). Manabí holdings had higher adjusted odds of all three preventive outcomes than holdings in the other Coast provinces (all *p* < 0.001), but not of self-reported disease (*p* = 0.493). Preventive engagement was strongest for the centrally organised foot-and-mouth disease campaign. These findings identify territorial and farm-level inequalities relevant to veterinary-service delivery and surveillance, without establishing animal-level coverage, confirmed prevalence, or causal effects.

## Simple Summary

Cattle health in Ecuador depends on effective vaccination, disease-control practices, and timely recognition of health problems, but participation may differ between farms and regions. This study used data from the 2025 national agricultural survey to examine reported participation in cattle vaccination and disease-control activities across continental Ecuador, with particular attention to Manabí Province. Participation was highest for the national foot-and-mouth disease vaccination campaign, while other cattle vaccination and disease-control activities were less frequent. Larger cattle herds were more likely to report all three health practices, and important regional differences were observed. Farms in Manabí reported greater participation in preventive activities than farms in the other coastal provinces. Larger herds were also more likely to report cattle disease, which may partly reflect better observation and reporting rather than greater disease occurrence alone. The findings can help veterinary authorities identify farms and territories that may need more accessible services, communication, and surveillance support as Ecuador moves from routine foot-and-mouth disease vaccination towards a stronger surveillance-based strategy.

## 1. Introduction

Foot-and-mouth disease (FMD) is a highly contagious transboundary viral disease affecting domestic and wild cloven-hoofed animals. Although mortality is generally low in adult cattle, infection causes substantial production losses through reduced milk yield, impaired growth and reproductive performance, and the costs associated with surveillance, vaccination, movement restrictions, outbreak control, and trade disruption. Because livestock movements and market restrictions can extend the consequences of an outbreak beyond affected holdings, FMD prevention requires coordinated surveillance, early detection and notification, movement control, biosecurity, and vaccination strategies appropriate to the epidemiological status of each country or zone [1,2,3].

South America has achieved substantial progress in FMD control through the Hemispheric Program for the Eradication of Foot-and-Mouth Disease and the progressive strengthening of national Veterinary Services [4,5]. In Ecuador, the final FMD case was recorded in August 2011. In 2015, continental Ecuador was officially recognised as an FMD-free zone where vaccination was practised, while the Galápagos Islands were recognised as an FMD-free zone without vaccination. This achievement followed the reorganisation of the national control programme, improvements in vaccination practices and quality assurance, livestock registration, animal-movement control, and serological surveillance [5].

Vaccination remained a central component of Ecuador’s sanitary strategy during the period covered by the study. The official campaign conducted from 14 February to 31 March 2025 aimed to immunise more than 4.7 million cattle and buffaloes throughout continental Ecuador [6]. Ecuador subsequently notified the World Organisation for Animal Health that routine FMD vaccination in cattle and buffaloes would cease from 29 January 2026, as part of the national plan to apply for recognition as FMD-free without vaccination in 2027 [7]. At the time of writing, however, continental Ecuador remained officially recognised as an FMD-free zone where vaccination was practised, while the Galápagos zone retained its recognition without vaccination [3]. The 2025 agricultural survey therefore provides a national baseline for examining reported holding-level participation during the year immediately preceding this policy transition. The epidemiological importance of maintaining broad and territorially coherent participation is reinforced by Ecuador’s cattle-movement structure. Analysis of national movement records from 2017 and 2018 identified a low-fragmentation network whose largest annual strongly connected component included more than 90% of parishes, indicating substantial potential for rapidly transmissible infections such as FMD to disseminate through livestock movements [8].

High national vaccination totals do not necessarily indicate uniform holding-level participation in a vaccination campaign. Participation may depend both on how veterinary services organize and deliver the programme and on the characteristics and decisions of cattle producers. Studies conducted in different livestock systems have linked vaccination behaviour or the intention to adopt disease-control measures with producer knowledge, perceptions of disease risk and vaccination, previous disease experience, herd characteristics, and production conditions [9,10,11,12,13]. Practical barriers, including limited service availability, distance to vaccination providers, affordability, and difficulties accessing veterinary assistance, may further influence whether livestock holdings use vaccination services [13].

Evidence from other predominantly small-scale livestock settings indicates that these barriers are context-dependent. FMD-control intentions differed across production systems in Ethiopia [9]; vaccination behaviour among traditional dairy farmers in Sri Lanka was associated with knowledge and farm characteristics [11]; vaccine availability, access to vaccinators, and information influenced vaccination decisions among small-scale livestock keepers in Myanmar [12]; and vaccination-service use in Ghana was constrained by service-access and farmer-level barriers [13]. These findings suggest that the relevant question is not only whether vaccination services exist nationally, but whether different types of holdings can access and use them consistently. Comparable nationally representative holding-level evidence linking vaccination and disease-control practices to farm, producer, and territorial characteristics has remained limited in Ecuador.

Nevertheless, holding-level evidence on the factors associated with participation in cattle vaccination and complementary disease-control practices in Ecuador remains limited. Official campaign totals provide aggregate numbers of vaccinated animals but do not identify which types of holdings were less likely to report participation in the campaign or the implementation of other preventive veterinary measures.

The 2025 Encuesta de Superficie y Producción Agropecuaria Continua (ESPAC) provides an appropriate data source for addressing this evidence gap. It combines holding-level cattle-health information with herd characteristics, production orientation, farm area, producer attributes, territorial location, and variables required to account for the complex survey design. The ESPAC methodology supports design-based estimation at national, regional, and provincial levels through the application of sampling strata, primary sampling units, and final expansion factors [14]. Manabí was selected a priori as the principal subnational focus because it accounted for an estimated 22.1% of Ecuador’s cattle population in 2025, the largest provincial share reported in the ESPAC technical bulletin [15].

Against this background, this study examined farm- and territorial-level differences in reported preventive cattle-health practices among holdings in continental Ecuador. The specific objectives were to: (i) estimate holding-level participation in the official FMD vaccination campaign conducted during the first semester of 2025; (ii) estimate the reported application of other cattle vaccines and implementation of disease-control practices; (iii) identify farm, producer, production-system, and territorial characteristics associated with each preventive outcome; and (iv) examine self-reported occurrence of a principal cattle disease as a secondary outcome. All estimates and regression models accounted for the final ESPAC expansion weights, sampling strata, and primary sampling units. Manabí was examined as a prespecified subnational domain because of its importance in Ecuadorian cattle production and its large share of the national cattle population [15]. The study provides a national pre-transition baseline for identifying inequalities in preventive participation and informing territorially differentiated veterinary surveillance, producer communication, and service delivery during the transition from routine vaccination to a surveillance-centred strategy.

## 2. Materials and Methods

The methodological approach comprised the definition of the study population and analytical samples, operationalisation of the holding-level outcomes and covariates, specification of the complex survey design, and design-adjusted descriptive and multivariable analyses. Particular attention was given to differences in the temporal reference periods of the ESPAC variables and to the distinction between reported holding-level practices and animal-level epidemiological measures. Data preparation and analysis followed a documented and reproducible workflow, while the original public-use databases were retained unchanged.

### 2.1. Study Design and Data Source

This study was a cross-sectional secondary analysis of anonymized public-use microdata from the 2025 Encuesta de Superficie y Producción Agropecuaria Continua (ESPAC), conducted by Ecuador’s Instituto Nacional de Estadística y Censos (INEC; National Institute of Statistics and Censuses). ESPAC is Ecuador’s principal official source of annual agricultural and livestock statistics. Its survey universe covers continental Ecuador, excluding densely populated urban areas and the Galápagos Islands, and its target population comprises land units used for agricultural or livestock production. The survey was designed to support estimation at national, regional, and provincial levels across the 23 provinces of continental Ecuador [14].

ESPAC 2025 was conducted during the final four months of the reference year using a multiple-frame sampling design that combined an area frame and a list frame. The area frame applied probabilistic stratified sampling of geographical sampling segments, whereas the list frame covered agricultural production units associated with products considered economically important or strategically relevant. Data were collected during field visits using interviewer-administered ESPAC questionnaires. Reference periods varied according to the variable and included the day of the interview, the period from 1 January 2025 to the interview date, and the 2025 reference year [14].

The public-use ESPAC 2025 release is organised into module-specific databases. This study used the cattle database (glnac2025) and the database of general characteristics of the agricultural producer (cgnac2025). Both databases contain the common unique variable Identificador, which was used for record linkage [16]. The cattle database supplied the veterinary outcomes, current herd size, production-orientation variables, and selected cattle-management information, whereas the producer database supplied the sociodemographic, residence, social-insurance, and digital-access variables used in the analysis. The linked analytical extract also contained total farm area (sup_ha). The original public-use files were retained unchanged, and all selection, linkage, transformation, and analysis procedures were documented to ensure traceability and reproducibility.

### 2.2. Study Population and Analytical Sample

The initial cattle-module dataset contained 10,715 records. Each record had a unique value of the survey variable Identificador, and no duplicate identifiers were detected. The cattle-module records were linked one-to-one to the database of general characteristics of the agricultural producer. All 10,715 records were successfully linked, and no cattle-module records were lost during the linkage procedure.

Eligibility at the initial data-preparation stage was not restricted to records with a positive current herd size because the veterinary outcomes and the herd-size covariate referred to different periods. Reported participation in the official FMD vaccination campaign referred to the campaign conducted during the first semester of 2025, and reported cattle disease referred to the period from 1 January 2025 to the interview date. Current herd size, by contrast, represented the number of cattle present on the holding on the interview date [17]. A holding could therefore provide valid vaccination or disease information even if no cattle remained at the time of the interview because animals may have been sold, transferred, slaughtered, or otherwise disposed of after the relevant veterinary event. Accordingly, the 14 records with a current herd size of zero and the 49 records with missing current herd size were retained in the traceable data-preparation dataset rather than excluded a priori. Zero herd size was treated as a valid value, whereas records with missing herd size were subsequently excluded from complete-case regression models requiring this covariate.

The final ESPAC expansion weight (fact_exp_fin) was used to determine eligibility for design-based estimation [14]. Of the 10,715 linked cattle-module records, 10,629 had a positive final expansion weight. The 86 records with a non-positive weight were retained in the traceable data-preparation dataset but excluded from all survey-weighted estimates and regression models. Among the positive-weight records, 41 were assigned to the official territorial category “undelimited zone” rather than to one of the 23 continental provinces. These records were retained for audit purposes but excluded from the principal territorial analyses because they could not be assigned consistently to a province, continental region, or the Manabí indicator used in the study.

The principal territorial descriptive domain comprised 10,588 positive-weight cattle holdings assigned to one of the 23 provinces of continental Ecuador. Outcome-specific denominators varied according to response validity. All 10,588 holdings had valid information for reported participation in the official FMD vaccination campaign, application of other cattle vaccines, and occurrence of any self-reported principal cattle disease. Valid information on disease-control practices was available for 10,576 holdings because 12 holdings had a missing or otherwise invalid response for this outcome.

The third outcome was the reported implementation of cattle disease-control activities (gl_controlenf) between 1 January 2025 and the interview date. Responses were coded as 1 when the producer reported having carried out disease-control activities and as 0 when no such activities were reported. The questionnaire did not specify the type, duration, frequency, or timing of the reported activities in relation to a disease event. This outcome was therefore interpreted conservatively as a self-reported disease-control practice and not as a direct measure of preventive biosecurity, treatment effectiveness, or access to a specific veterinary service [17,18].

Self-reported cattle disease was examined as a secondary outcome using the variable identifying the principal disease reported to have affected the cattle between 1 January 2025 and the interview date (gl_prinenfe). The original response categories were milk fever, mastitis, tympanism, brucellosis, vesicular diseases, another disease, and no disease. A binary analytical variable (any_reported_disease) was coded as 1 for any of the first six categories and as 0 for “no disease”; the original categories were retained for descriptive analysis. Because these responses were producer-reported and were not supported by clinical examination, laboratory confirmation, or official outbreak investigation, “vesicular diseases” was not interpreted as confirmed FMD. Brucellosis and the other named conditions were likewise treated as reported disease categories rather than confirmed diagnoses [17,18].

The cattle module also recorded the number of animals reportedly vaccinated during the official FMD campaign (gl_numvacunado) and the number reportedly affected by disease (gl_numenfermo). These counts were retained for traceability and data-quality assessment but were not used to calculate animal-level ratios. The vaccination count referred to the first semester of 2025, the disease count referred to the period from 1 January 2025 to the interview date, and current herd size referred to the interview date. Purchases, sales, births, deaths, slaughter, transfers, and other herd changes between these periods could therefore make the numerators incompatible with the current herd-size denominator. Consequently, neither animal-level vaccination coverage nor animal-level disease prevalence was estimated from these variables [17,18].

Valid information on disease-control practices was available for 10,576 holdings because 12 holdings had a missing or otherwise invalid response for this outcome.

### 2.3. Outcome Variables

Four holding-level outcomes were examined: (i) reported participation in the official foot-and-mouth disease (FMD) vaccination campaign; (ii) reported application of any cattle vaccine other than the official FMD vaccine; (iii) reported implementation of cattle disease-control activities; and (iv) occurrence of any self-reported principal cattle disease. The original ESPAC response codes were retained in the traceable working dataset, and binary analytical variables were subsequently derived using prespecified coding rules. Where present, missing or invalid responses were retained as missing and were not assigned to either binary category.

The principal outcome was reported holding-level participation in the official FMD vaccination campaign (gl_vacuna). The corresponding survey question asked whether the cattle under the producer’s responsibility had been vaccinated against FMD through the AGROCALIDAD programme during the first semester of 2025, defined in the interviewer manual as the period from January to June. Responses coded as 1 (“yes”) were assigned a value of 1, and responses coded as 2 (“no”) were assigned a value of 0. This outcome represents producer-reported holding-level participation in the official campaign and should not be interpreted as an independently verified animal-level vaccination record or as a measure of animal-level vaccination coverage [17,18].

The second preventive outcome was the reported application of a cattle vaccine other than the official FMD vaccine supplied through the AGROCALIDAD programme (gl_aplico_otra_vacuna). The questionnaire asked whether any other vaccine had been applied to the cattle between 1 January 2025 and the interview date. Affirmative responses were coded as 1 and negative responses as 0. The survey also recorded the type of entity that supplied or administered the additional vaccine; however, this information was conditional on an affirmative response and was not analysed as a separate outcome in the principal regression models. The resulting variable therefore identifies reported holding-level application of another cattle vaccine but does not indicate the number or proportion of animals vaccinated [17,18].

The third outcome was the reported implementation of cattle disease-control activities (gl_controlenf) between 1 January 2025 and the interview date. Responses were coded as 1 when the producer reported having carried out disease-control activities and as 0 when no such activities were reported. The questionnaire did not specify the type, duration, frequency, or timing of the reported activities in relation to a disease event. This outcome was therefore interpreted conservatively as a self-reported disease-control practice and not as a direct measure of preventive biosecurity, treatment effectiveness, or access to a specific veterinary service [17,18].

Self-reported cattle disease was examined as a secondary outcome using the variable identifying the principal disease reported to have affected the cattle between 1 January 2025 and the interview date (gl_prinenfe). The original response categories were milk fever, mastitis, tympanism, brucellosis, vesicular diseases, another disease, and no disease. A binary analytical variable (any_reported_disease) was coded as 1 when any of the first six categories was reported and as 0 when the response was “no disease”. The original disease categories were retained for descriptive analysis. Because the responses were producer-reported and were not supported by clinical examination, laboratory confirmation, or official outbreak investigation, the category “vesicular diseases” was not interpreted as confirmed FMD. Brucellosis and the other named conditions were likewise treated as reported disease categories rather than confirmed veterinary diagnoses [17,18].

The binary variable was used only as a secondary holding-level indicator distinguishing any reported principal cattle-health condition from no reported principal disease. The six positive categories were not assumed to constitute a biologically homogeneous disease endpoint. Disease-specific multivariable models were not fitted because several individual categories were sparse and because none represented clinically or laboratory-verified diagnoses. The original categories were instead retained for separate descriptive presentation.

The cattle module also recorded the number of animals reportedly vaccinated during the official FMD campaign (gl_numvacunado) and the number reportedly affected by disease (gl_numenfermo). These counts were retained for traceability and data-quality assessment but were not used to calculate animal-level ratios or as outcomes in the principal regression models. The vaccination count referred to the first semester of 2025, the disease count referred to the period from 1 January 2025 to the interview date, and current herd size referred to the number of cattle present on the interview date. Purchases, sales, births, deaths, slaughter, transfers, and other herd changes occurring between these periods could therefore make the numerators incompatible with the current herd-size denominator. Consequently, neither animal-level vaccination coverage nor animal-level disease prevalence was estimated from these variables [17,18]. The reference periods could not be harmonised retrospectively because they were defined by the official ESPAC questionnaire. Analyses therefore preserved each variable’s specified reference period rather than imposing an artificial common window. Cross-outcome comparisons should be interpreted as comparisons of holding-level reporting within the 2025 survey context, not as rates measured over identical observation periods, and no temporal causal ordering among the outcomes is inferred.

### 2.4. Farm- and Producer-Level Covariates

A common prespecified set of farm- and producer-level covariates was used in all four regression models to facilitate comparison of the factors associated with the different veterinary outcomes. Covariates were selected according to their conceptual relevance to herd structure, production characteristics, producer attributes, access to information, and potential access to services, together with their availability and consistency in the ESPAC databases. The same covariate structure was retained across the four models; variables were not selected separately for each outcome on the basis of univariable statistical significance.

Farm scale was represented by current herd size and total farm area. Current herd size was calculated as the total number of owned and non-owned cattle present on the holding on the interview date (*gl_k801*). Total farm area corresponded to the combined surface area, expressed in hectares, of all land parcels under the responsibility of the agricultural producer or responsible person, both within and outside the sampled segment (*sup_ha*) [17,18]. Current herd size and farm area showed strongly right-skewed distributions and were transformed as log(1 + x) before inclusion in the regression models. Zero was a valid value for current herd size and was retained by this transformation. Model coefficients for these variables were interpreted per one-unit increase in log(1 + current herd size) or log(1 + farm area), rather than per additional animal or hectare.

Cattle-production orientation was derived from the numbers of owned and non-owned cattle classified by ESPAC as intended for milk, meat, or dual-purpose production. Milk-oriented cattle were calculated from *gl_propleche* and *gl_ajenleche*, meat-oriented cattle from *gl_propcarne and gl_ajencarne*, and dual-purpose cattle from *gl_propdoblep* and *gl_ajendoblep*. For each holding, the owned and non-owned counts were summed within each orientation, and the holding was assigned to the category with the largest total. Holdings with equal positive maximum totals in more than one category were classified as mixed, whereas holdings with no cattle assigned to any of the three orientations were classified as not classified. The resulting categories were milk, meat, dual purpose, mixed, and not classified. Dual-purpose production was used as the reference category in the regression models [17,18].

Producer characteristics referred to the agricultural producer or responsible person identified in the ESPAC database. Age was obtained from *cg_k101*, measured in completed years, and rescaled in 10-year units to improve the interpretability of the regression coefficients. Producer sex was derived from *cg_k100* and represented by a binary indicator comparing women with men; men constituted the reference category. Formal education was derived from instruction and grouped into primary education, secondary education, higher education, and no formal education. The higher-education category combined university and postgraduate education because comparatively few producers reported postgraduate education. Primary education was used as the reference category. The single record coded as education not reported was excluded from regression models requiring this covariate [17,18].

Digital access was represented by a composite score constructed from three binary variables indicating the availability of fixed internet service (*cg_dispo_internet*), a computer or laptop (*cg_dispo_comp*), and a smartphone (*cg_dispo_tlfint*) at the agricultural production unit. Each affirmative response contributed one point, producing a score ranging from 0, when none of the three resources was available, to 3, when all three were available. The score was calculated only when valid responses were available for all three components. It was interpreted as an indicator of access to digital resources and not as a measure of digital literacy, frequency of technology use, internet connectivity quality, or access to veterinary information. Residence on the holding was derived from *cg_vive_predio_pr* and coded as 1 when the agricultural producer or responsible person reported living on the holding and as 0 otherwise. Affiliation with the Seguro Social Campesino (Rural Social Insurance Scheme) was derived from cg_segsoccam and coded as 1 for reported affiliation and as 0 for no affiliation. Absence of residence on the holding and no affiliation with the Rural Social Insurance Scheme were used as the corresponding reference categories [17,18].

Variables describing the use of specific veterinary, vaccination, antiparasitic, and other animal-health services were not included as explanatory covariates in the principal models. These variables were temporally and conceptually close to the vaccination and disease-control outcomes and could represent components, consequences, or intermediate variables of the practices under investigation. Their inclusion could therefore have resulted in overadjustment or ambiguous coefficient interpretation. Detailed variables concerning breed, reproduction, feeding, and veterinary-service use were retained in the traceable working dataset but were not included in the principal multivariable models.

Producer income, detailed on-farm biosecurity infrastructure, and distance or travel time to veterinary service points were considered conceptually relevant but were not available in the ESPAC 2025 public-use analytical files. Exact farm coordinates were also not released, precluding derivation of holding-to-service distances. These variables were therefore not approximated post hoc using unvalidated proxies.

### 2.5. Territorial Classification and Prespecified Focus on Manabí

Territorial location was determined using the ESPAC province variable (*ual_prov*). The principal territorial domain comprised cattle holdings assigned to one of the 23 provinces of continental Ecuador. Positive-weight records assigned to the official category “undelimited zone” were excluded from this domain because they could not be assigned consistently to a province, analytical region, or the Manabí indicator. Galápagos was outside the geographical scope of the ESPAC 2025 survey and was therefore not represented in the analytical dataset.

**Figure 1:**
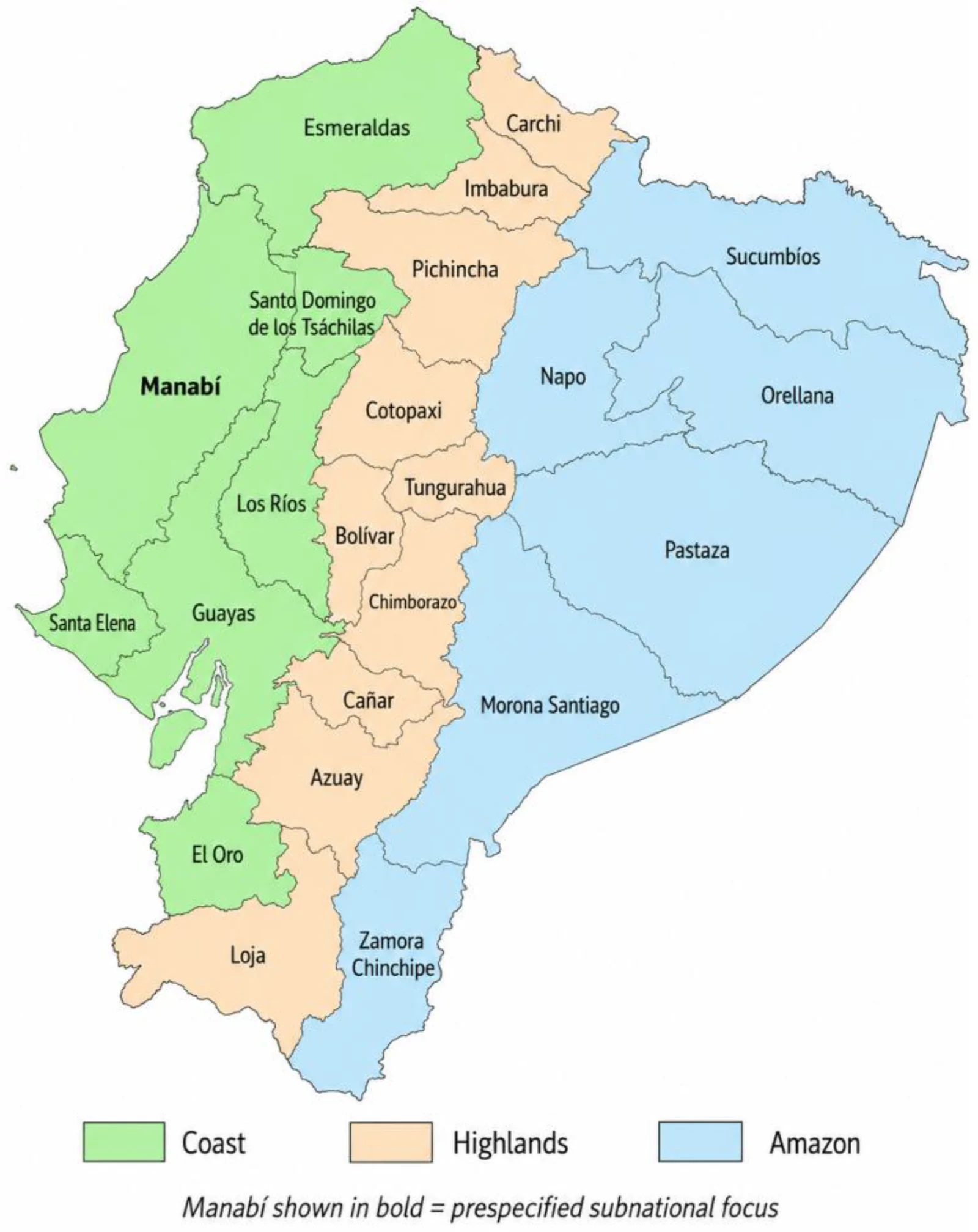
Territorial classification of the 23 continental provinces included in the ESPAC 2025 analytical domain. Provinces were grouped into the Coast, Highlands, and Amazon regions. Manabí remained within the Coast and was additionally represented by a prespecified indicator in the adjusted models. The “undelimited zone” and Galápagos were not included in the analytical territorial classification. Source: Authors’ elaboration based on ESPAC 2025 [14].

For the regression analyses, the 23 continental provinces were grouped into three geographical regions. The Coast comprised El Oro, Esmeraldas, Guayas, Los Ríos, Manabí, Santo Domingo de los Tsáchilas, and Santa Elena. The Highlands comprised Azuay, Bolívar, Cañar, Carchi, Cotopaxi, Chimborazo, Imbabura, Loja, Pichincha, and Tungurahua. The Amazon region comprised Morona Santiago, Napo, Pastaza, Zamora Chinchipe, Sucumbíos, and Orellana. Region was entered as a categorical covariate, with the Coast used as the reference category. This classification is shown in Figure 1.

Manabí was selected a priori as the principal subnational focus because of its importance in Ecuadorian cattle production and not because of the results observed in the present analysis. Official ESPAC estimates indicated that Manabí accounted for approximately 22.1% of Ecuador’s cattle population in 2025, representing the largest provincial share reported for continental Ecuador [15]. The original cattle-module database contained 1423 records from Manabí, of which 1394 had a positive final expansion weight and were included in the principal Manabí descriptive domain.

A binary Manabí indicator was included in each regression model and coded as 1 for holdings located in province code 13 and as 0 for holdings in the other 22 continental provinces. Manabí remained classified within the Coast region in the regional covariate. Because geographical region and the Manabí indicator were entered simultaneously, the Manabí coefficient represents the adjusted contrast between holdings in Manabí and holdings in the other Coast provinces. It does not represent a comparison between Manabí and all other provinces of Ecuador.

Design-adjusted descriptive estimates were additionally calculated for Manabí. A separate Manabí-only multivariable model was not fitted because the principal inferential objective was to estimate associations across the complete 23-province continental domain while assessing Manabí through the prespecified indicator. Santa Elena was retained within the Coast category and in the province-level descriptive analyses. However, no Santa Elena-specific regression model or province indicator was fitted because only 26 eligible cattle holdings were available and all 26 reported participation in each of the three preventive outcomes, resulting in no within-province outcome variation.

### 2.6. Complex Survey Design and Weighting

All design-adjusted descriptive estimates and regression models accounted for the complex sampling design of ESPAC 2025. The survey used a multiple-frame design combining an area frame and a list frame. Within the area frame, geographical sampling segments were selected through stratified cluster sampling. The list frame included agricultural production units associated with economically important or strategically relevant products and operated as a census-like component. The analytical design incorporated the final expansion weight, sampling strata, and primary sampling units (PSUs) available in or derived from the public-use data [14].

The final ESPAC expansion weight *(fact_exp_fin*) was used as the survey weight. For units selected through the area frame, this weight originated from the inverse probability of segment selection and incorporated the adjustments applied by INEC for incomplete coverage, partial investigation, segment subdivision, provincial surface correction, and differences between mapped and field-reported land area. Units included in the list frame received an expansion weight of 1 because the eligible units in that frame were investigated as certainty units [14]. Only records with a positive final expansion weight were eligible for design-based estimation. Of the 10,715 linked cattle-module records, 10,629 had a positive weight; the remaining 86 records were retained in the traceable data-preparation dataset but excluded from survey-weighted estimates and regression models.

Analytical strata were defined by combining the province code (*ual_prov*) with the ESPAC stratum variable (*ual_estr*), because the stratum codes were defined within provinces and were not necessarily unique across the national dataset. PSUs were defined by combining province, stratum, and sampling-segment identifiers (*ual_prov*, *ual_estr*, and *ual_segm*). These composite identifiers ensured that strata and PSUs were uniquely identified across provinces. Among the 10,629 positive-weight records, the resulting design contained 142 strata and 3600 PSUs. After exclusion of the 41 records assigned to the “undelimited zone” category, the principal 23-province territorial domain contained 139 strata and 3586 PSUs.

Design-adjusted proportions were estimated as the ratio of the weighted total of holdings reporting the outcome to the weighted total of holdings with a valid response for that outcome. Standard errors were estimated using first-order Taylor linearisation, with linearised contributions aggregated at the PSU level within sampling strata. This procedure accounted for unequal selection probabilities, stratification, and within-PSU dependence and avoided treating the ESPAC observations as a simple random sample [19].

The positive-weight survey design contained 58 strata represented by only one observed PSU. Because within-stratum variability cannot be estimated directly when a stratum contains a single observed PSU, the principal variance specification centred the contribution of each singleton PSU relative to the overall sample rather than assigning it zero variance. A sensitivity analysis applied an alternative specification in which each singleton stratum was assigned the average variance contribution estimated from strata containing more than one PSU. An association was considered design-robust when its direction, substantive interpretation, and statistical evidence remained consistent under both singleton-stratum specifications.

Design degrees of freedom were calculated as the number of observed PSUs minus the number of observed strata. Confidence intervals and hypothesis tests were based on the corresponding Student’s t distribution rather than the standard normal distribution. Finite-population corrections were not applied because the first-stage population counts required for their specification were not available as analytical variables in the public-use cattle and producer databases. The final expansion weights were therefore used to recover population representation, while the stratum and PSU identifiers were used to estimate sampling variability.

Estimates for Manabí, Santa Elena, and the other individual provinces were calculated as survey-domain estimates rather than as estimates from independently defined subsamples. The complete 23-province survey-design structure was retained, with records outside the domain under consideration contributing zero to the corresponding domain total. This approach preserved the original strata and PSUs for variance estimation and avoided treating a territorial subset as though it had been selected through an independent sampling design [19].

### 2.7. Statistical Analysis

The analysis comprised three stages: data-quality assessment, design-adjusted descriptive estimation, and survey-weighted multivariable modelling. Data preparation, variable derivation, descriptive estimation, and inferential analyses were conducted through a reproducible Python workflow using pandas, NumPy, Patsy, SciPy, and statsmodels (Python version 3.12.13, pandas version 2.2.2, NumPy version 2.0.2, Patsy version 1.0.2, SciPy version 1.16.3, and statsmodels version 0.14.6.). Custom analytical routines were used to incorporate the final expansion weights, sampling strata, PSU clustering, Taylor-linearised variance estimation, and the singleton-stratum procedures described in Section 2.6.

First, all analytical variables were examined for duplicate identifiers, invalid response codes, missing values, non-positive expansion weights, inconsistent territorial classifications, and incompatibilities arising from different temporal reference periods. Unweighted counts were used to describe the observed sample, whereas proportions intended to represent the population of cattle holdings were calculated using the final ESPAC expansion weight. Animal-level vaccination coverage and disease prevalence were not estimated from the reported numbers of vaccinated or affected cattle because those counts and current herd size referred to different time points.

Design-adjusted proportions and 95% confidence intervals were estimated for the four holding-level outcomes: reported participation in the official FMD vaccination campaign, reported application of another cattle vaccine, reported implementation of disease-control activities, and occurrence of any self-reported principal cattle disease. Estimates for the complete 23-province continental domain were calculated from all eligible holdings. Manabí, Santa Elena, and the remaining individual provinces were analysed as survey domains while retaining the complete territorial survey-design structure, as described in Section 2.6.

Associations between the prespecified farm-, producer-, production-system-, and territorial covariates and each binary outcome were examined using four separate survey-weighted logistic pseudo-likelihood models with a logit link. Within each outcome-specific model, positive expansion weights were divided by their mean before model fitting to preserve their relative contribution while improving numerical stability. Model-based score contributions were aggregated by PSU within sampling strata, and the design-adjusted sandwich covariance matrix was calculated using the Taylor-linearisation and singleton-stratum procedures described in Section 2.6 [19].

The same prespecified covariate structure was entered simultaneously in all four models: log(1 + current herd size), log(1 + total farm area), producer age in 10-year units, producer sex, formal education, digital-access score, residence on the holding, affiliation with the Rural Social Insurance Scheme, cattle-production orientation, geographical region, and the Manabí indicator. The reference categories were male producer, primary education, dual-purpose production, no residence on the holding, no affiliation with the Rural Social Insurance Scheme, and the Coast region. Because geographical region and the Manabí indicator were included simultaneously, the Manabí coefficient represents the adjusted contrast between holdings in Manabí and holdings in the other Coast provinces.

No stepwise, automated, or outcome-dependent variable-selection procedure was applied. The common adjustment set was retained to support comparison across outcomes and to avoid selecting covariates solely on the basis of sample-specific univariable associations. Outcome-specific complete-case samples were used, and a holding was excluded from a particular model only when the corresponding outcome or one of the covariates required for that model was missing. The unweighted analytical sample sizes were 10,538 holdings for the official FMD vaccination model, 10,538 for the other-cattle-vaccination model, 10,526 for the disease-control-practice model, and 10,538 for the self-reported-disease model. No statistical imputation was performed.

Regression coefficients were exponentiated and reported as adjusted odds ratios (aORs) with 95% confidence intervals. Standard errors were obtained from the design-adjusted covariance estimator, and confidence intervals and hypothesis tests used the design degrees of freedom defined as the number of observed PSUs minus the number of observed strata. Two-sided *p*-values below 0.05 were considered evidence of statistical association. No formal correction for multiple comparisons was applied; interpretation considered effect magnitude, direction, confidence-interval width, consistency across related outcomes, epidemiological plausibility, and stability under the alternative singleton-stratum variance specification.

All models were interpreted as associational rather than causal. Associations with self-reported disease may reflect differences in disease occurrence, recognition, observation, access to information, or willingness to report. Associations with vaccination and disease-control practices may reflect both producer and holding characteristics and territorial differences in veterinary-service delivery. The adjusted odds ratios therefore describe conditional associations within the observed cross-sectional survey data and should not be interpreted as individual-level causal effects.

### 2.8. Sensitivity and Data-Quality Analyses

A structured data-quality assessment was completed before design-adjusted descriptive estimation and multivariable modelling. The assessment examined identifier uniqueness, linkage completeness, response-code validity, missingness, expansion-weight eligibility, territorial classification, temporal compatibility among variables, and the construction of derived analytical variables. All checks were performed using the traceable data-preparation dataset, while the original public-use ESPAC files were retained unchanged.

The uniqueness of the common survey variable Identificador was verified in the cattle and producer databases before linkage. No duplicate identifiers were detected in the cattle database, and all 10,715 cattle-module records were linked one-to-one to the corresponding producer-characteristics records. Province, stratum, and sampling-segment codes were checked before the composite analytical stratum and PSU identifiers were constructed. The 86 records with a non-positive final expansion weight were retained for audit purposes but excluded from all survey-weighted estimates and regression models. The 41 positive-weight records assigned to the official “undelimited zone” category were also retained for traceability but excluded from analyses requiring assignment to one of the 23 continental provinces.

Temporal compatibility was assessed particularly for current herd size, the reported number of animals vaccinated during the official FMD campaign, and the reported number of animals affected by disease. Current herd size referred to the interview date, the vaccination count referred to the first semester of 2025, and the disease count referred to the period from 1 January 2025 to the interview date. Holdings in which the reported number vaccinated or affected exceeded the herd size present on the interview date were not automatically classified as data errors because purchases, sales, births, deaths, slaughter, transfers, and other herd changes could have occurred between the respective reference periods. The original counts were therefore retained, but neither vaccination coverage nor disease prevalence was calculated using current herd size as the denominator.

Records with a current herd size of zero were distinguished from records with missing herd size. The 14 zero values were treated as valid because a holding could have reported vaccination or disease events occurring earlier in 2025 even if no cattle remained on the interview date. The 49 records with missing current herd size were retained in the data-preparation dataset and could contribute to analyses that did not require this covariate, but they were excluded from complete-case regression models because current herd size was included in all four model specifications.

No statistical imputation was performed for missing outcomes or covariates. Descriptive denominators were defined separately for each outcome according to response validity, and each regression model used an outcome-specific complete-case sample. A holding was excluded only from analyses requiring the variable for which information was missing; missingness in a variable not used in a particular analysis did not determine eligibility for that analysis. The number of valid observations and the unweighted complete-case sample size were recorded separately for each outcome and model.

Because the four outcomes were based on single interviewer-administered producer reports and the public-use data contained no repeated measurements, clinical validation sample, or linked administrative gold standard, formal test–retest reliability or measurement-error correction could not be performed. As a limited sensitivity analysis related to opportunity for direct observation, design-adjusted outcome proportions were additionally estimated after stratifying holdings by whether the producer or responsible person lived on the holding. This analysis was used only to assess descriptive stability across different observation contexts and was not interpreted as a correction for recall or cognitive bias.

Potential collinearity within the common covariate structure was examined using approximate variance inflation factors calculated from the analytical model matrix. VIF values ranged from 1.02 to 3.62. The highest values were observed for log-transformed farm area (3.62), log-transformed current herd size (3.27), Highlands versus Coast (2.57), the Manabí indicator (1.85), and Amazon versus Coast (1.48); all remaining model terms had VIF values below 1.46. These diagnostics did not indicate substantial redundancy among the prespecified predictors and were not used as an automated variable-selection criterion. The same covariate set was therefore retained across the four models.

The principal variance specification used first-order Taylor linearisation and retained the score-based contribution of each singleton PSU rather than assigning the corresponding stratum zero variance. As a sensitivity analysis, all design-adjusted descriptive estimates and survey-weighted regression models were repeated using an alternative procedure in which each singleton stratum was assigned the average variance contribution estimated from strata containing more than one observed PSU [19]. Because the alternative procedure modified only the variance estimator, point estimates remained unchanged, whereas standard errors, confidence intervals, and *p*-values could differ.

For descriptive estimates, sensitivity was evaluated by comparing confidence intervals under the two singleton-stratum procedures. For regression models, an association was considered design-robust when its direction, substantive interpretation, and statistical evidence were consistent under both variance specifications. Associations that retained a similar point estimate but crossed the conventional significance threshold under the alternative variance procedure were classified as variance-sensitive rather than as contradictory findings.

Additional interpretative safeguards were applied to small territorial domains. Santa Elena was retained in the province-level descriptive analysis, but its estimates were not interpreted as stable provincial evidence because only 26 eligible cattle holdings were observed. All 26 holdings reported participation in each of the three preventive outcomes, producing estimates of 100% and zero estimated standard error under the observed sample design. These values were therefore presented together with the unweighted sample size and an explicit caution that the apparent precision reflected the absence of observed variation in a small sample rather than certainty about the provincial population.

All analytical decisions, including sample restrictions, variable recoding, derived variables, data-quality flags, model-specific missingness, collinearity diagnostics, and singleton-stratum sensitivity procedures, were documented in the reproducibility package. The model-ready dataset, analytical scripts, coefficient tables, design-adjusted estimates, sensitivity outputs, variable definitions, and metadata were retained to support verification of the analytical workflow.

### 2.9. Ethical Considerations

This study was a secondary analysis of anonymised public-use microdata collected and released by Ecuador’s Instituto Nacional de Estadística y Censos (INEC). The authors did not participate in the original data collection and had no direct contact with agricultural producers. The public-use files did not provide respondent names, contact details, exact farm coordinates, or other direct identifiers that would allow the authors to identify individual respondents or cattle holdings.

The study did not involve the recruitment of human participants, the collection of new personal information, experimental procedures, clinical examinations, biological sampling, veterinary interventions, or the direct observation, restraint, treatment, or handling of live animals. All cattle-health information analysed in the study had been collected previously through the official ESPAC survey and was examined only as anonymised secondary survey data.

No new institutional ethics approval or individual informed consent was obtained specifically for this secondary analysis because the study used anonymised public-use data and involved neither direct human-participant research nor direct animal experimentation. The data were used solely for scientific analysis, and the original public-use files were retained unchanged. Results were reported only in aggregate form, and no record-level information or combinations of variables intended to identify individual producers or cattle holdings were disclosed.

## 3. Results

The results are presented in four parts. First, the construction of the analytical sample and the survey-design structure are described. Second, design-adjusted estimates of the four holding-level outcomes are reported for the principal continental domain and the prespecified territorial domains. Third, the survey-weighted multivariable models are presented for each outcome. Finally, the stability of the estimates and adjusted associations under the alternative singleton-stratum variance specification is examined.

### 3.1. Analytical Sample Construction and Survey-Design Structure

The original ESPAC 2025 cattle-module database contained 10,715 records with unique values of Identificador. All records were successfully linked one-to-one to the corresponding producer-characteristics records, and no duplicate cattle identifiers were detected. Of the 10,715 linked records, 10,629 had a positive final expansion weight and were eligible for design-based estimation. The remaining 86 records had non-positive weights and were retained in the traceable data-preparation dataset but excluded from all survey-weighted estimates and regression models.

Among the positive-weight records, 41 were assigned to the official “undelimited zone” category and could not be classified consistently into one of the 23 continental provinces. After their exclusion from the principal territorial analyses, the principal continental analytical domain comprised 10,588 cattle holdings.

All 10,588 holdings had valid information for reported participation in the official FMD vaccination campaign, application of other cattle vaccines, and occurrence of any self-reported principal cattle disease. Valid information on disease-control activities was available for 10,576 holdings because 12 holdings had a missing or otherwise invalid response for this outcome.

The principal continental survey-design domain comprised 139 sampling strata and 3586 PSUs. Across the complete positive-weight design, 58 strata were represented by only one observed PSU and were handled using the principal singleton-stratum variance specification. The Manabí descriptive domain comprised 1394 positive-weight cattle holdings distributed across 404 PSUs and nine strata. Santa Elena comprised 26 positive-weight holdings distributed across 14 PSUs and four strata. The construction of the analytical sample and the principal survey-design characteristics are summarised in Table 1.

**Figure 2:**
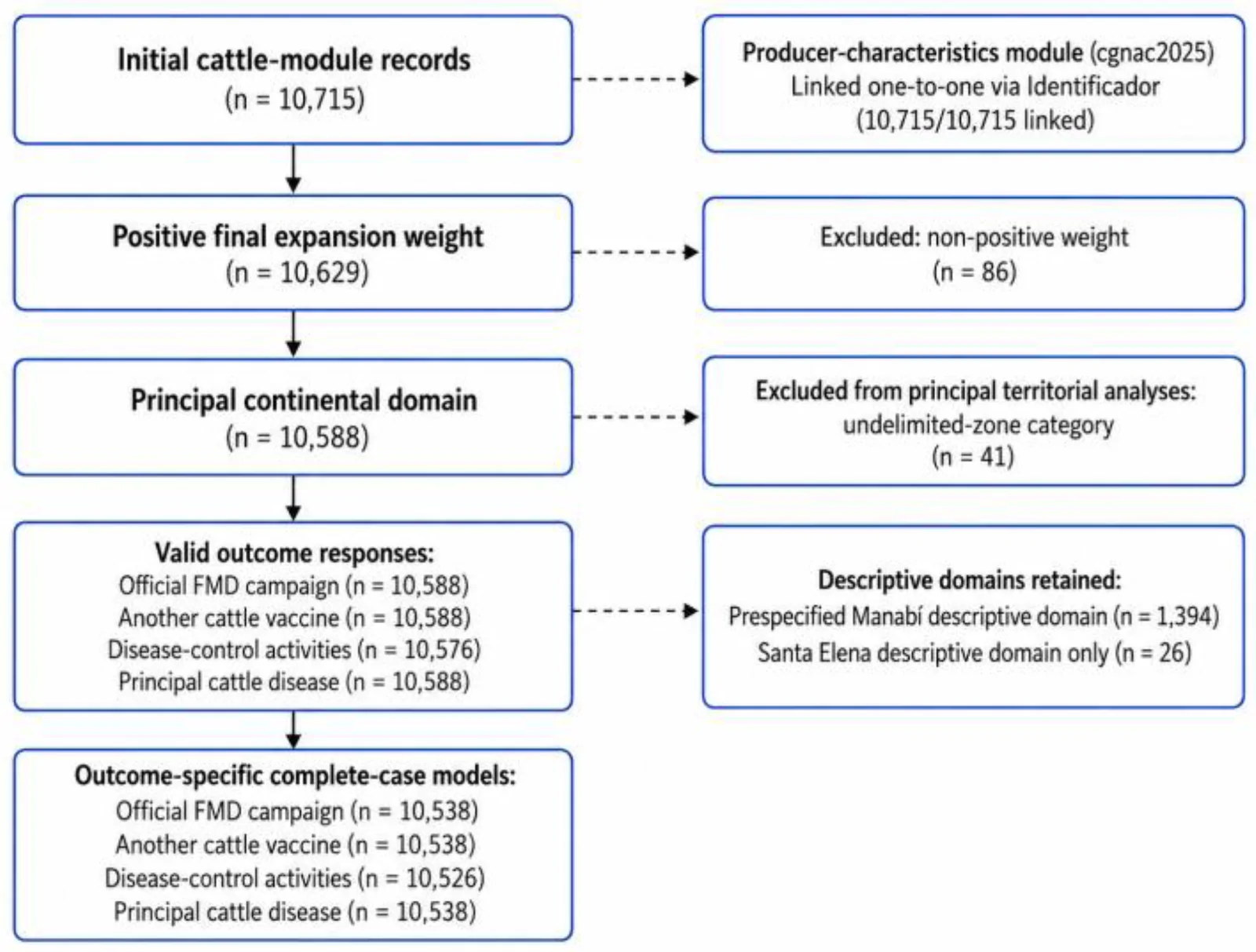
Flowchart of data cleaning, record linkage, survey-weight eligibility, territorial selection, and construction of the outcome-specific analytical samples. The original ESPAC 2025 cattle-module database contained 10,715 records, all of which were linked one-to-one to the producer-characteristics database using Identificador. Of these, 10,629 records had a positive final expansion weight. After excluding 41 positive-weight records assigned to the official “undelimited zone” category, the principal continental analytical domain comprised 10,588 cattle holdings. Outcome-specific valid samples and complete-case regression samples are shown in the final stages of the flowchart. FMD, foot-and-mouth disease.

The data-cleaning, record-linkage, survey-weight eligibility, territorial selection, and outcome-specific analytical steps are summarised in Figure 2.

### 3.2. Design-Adjusted Estimates of Vaccination, Disease-Control Practices, and Reported Disease

Within the principal continental domain, an estimated 86.38% of cattle holdings reported participation in the official FMD vaccination campaign (95% CI: 85.10–87.66%). The estimated proportion reporting application of another cattle vaccine was 55.96% (95% CI: 53.99–57.92%), and 64.74% reported implementation of disease-control activities (95% CI: 62.84–66.64%). The estimated proportion reporting at least one principal cattle disease was 11.22% (95% CI: 10.25–12.19%).

Across the three cattle-health-practice outcomes, a clear descriptive ordering was observed: official FMD campaign participation was highest, followed by disease-control activities and application of another cattle vaccine. Self-reported principal cattle disease was less frequent (11.22%), but it represents a different outcome construct and should not be interpreted as part of the same preventive-participation hierarchy.

Regional domain estimates showed substantial variation in the three cattle-health-practice outcomes. Reported participation in the official FMD campaign was 92.57% in the Coast, 84.94% in the Highlands, and 82.92% in the Amazon. Application of another cattle vaccine was 79.15%, 50.37%, and 45.82%, respectively, while reported disease-control activities were 84.48%, 59.60%, and 61.49%. In contrast, self-reported principal cattle disease was lowest in the Coast (7.91%), followed by the Highlands (11.90%) and the Amazon (14.27%). These are descriptive design-adjusted domain estimates and do not represent covariate-adjusted regional effects.

In Manabí, the estimated proportions reporting each of the three preventive outcomes were higher than the corresponding estimates for the principal continental domain. Reported participation in the official FMD vaccination campaign was estimated at 95.72% (95% CI: 93.76–97.67%), application of another cattle vaccine at 85.99% (95% CI: 82.94–89.04%), and implementation of disease-control activities at 91.31% (95% CI: 88.75–93.88%). The estimated proportion reporting at least one principal cattle disease was 8.11% (95% CI: 5.66–10.56%). These unadjusted domain estimates describe the observed territorial distributions and do not establish that location in Manabí independently caused the differences.

**Figure 3:**
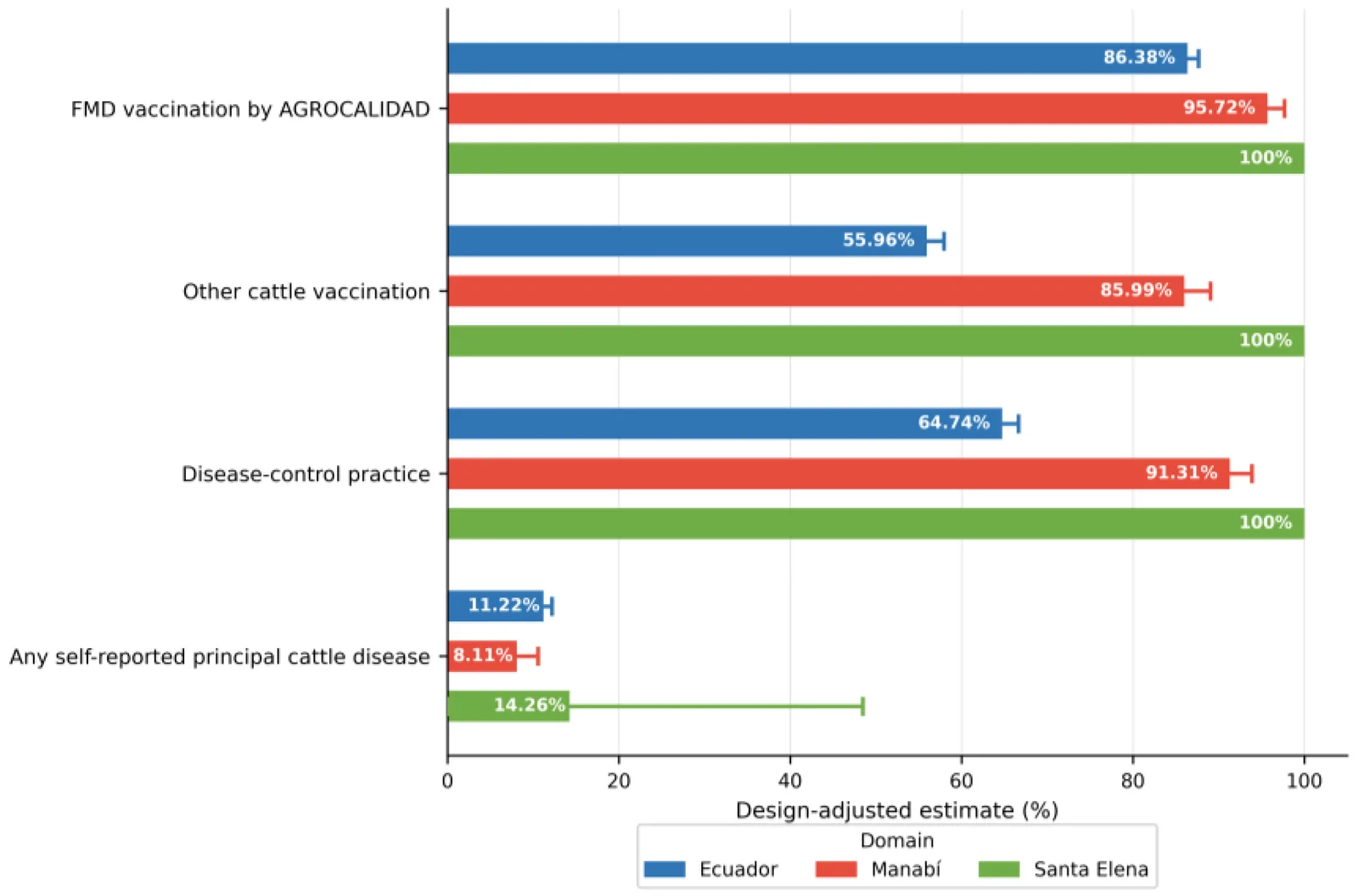
Design-adjusted holding-level estimates of official FMD campaign participation, application of another cattle vaccine, implementation of disease-control activities, and self-reported principal cattle disease in the principal continental domain, Manabí, and Santa Elena. Note: Points represent design-adjusted holding-level percentages, and horizontal error bars represent 95% confidence intervals. Estimates for official FMD campaign participation and application of another cattle vaccine refer to the proportion of holdings reporting participation or application and should not be interpreted as animal-level vaccination coverage. The self-reported principal-disease estimate should not be interpreted as animal-level or pathogen-specific prevalence. The Santa Elena estimates for the three cattle-health-practice outcomes were 100.0% and therefore had no visible sampling variation in the plotted point estimates. The unweighted Santa Elena domain comprised 26 holdings, and the 95% confidence interval for self-reported principal cattle disease was 0.00–48.48%. FMD, foot-and-mouth disease.

All 26 sampled cattle holdings in Santa Elena reported participation in the official FMD vaccination campaign, application of another cattle vaccine, and implementation of disease-control activities. The resulting design-adjusted estimates were 100.00%, with zero estimated standard error because no variation in these outcomes was observed in the sample. These values should not be interpreted as evidence of complete preventive participation throughout the provincial cattle-holding population. The estimated proportion reporting at least one principal cattle disease was 14.26%, but the 95% confidence interval was extremely wide, ranging from 0.00% to 44.37%. The complete estimates are presented in Table 2, and Figure 3 provides a visual comparison of the three geographical domains.

In the residence-stratified sensitivity analysis, the ordering of the three cattle-health-practice outcomes was unchanged in both subgroups. Among producers living on the holding (*n* = 4848), the design-adjusted estimates were 85.01% for official FMD campaign participation, 56.22% for other vaccination, 65.83% for disease-control activities, and 12.09% for self-reported principal disease. Corresponding estimates among producers not living on the holding (*n* = 5740) were 87.90%, 55.67%, 63.54%, and 10.25%, respectively. The self-reported-disease estimates were 12.09% (95% CI: 10.66–13.53%) and 10.25% (95% CI: 9.14–11.37%), respectively. These descriptive differences may be compatible with variation in observation opportunity but do not quantify or correct recall or reporting bias.

### 3.3. Factors Associated with Participation in the Official Foot-and-Mouth Disease Vaccination Campaign

The survey-weighted multivariable model included 10,538 cattle holdings with complete information for the outcome and all prespecified covariates. Adjusted associations were observed for formal education, cattle-production orientation, current herd size, total farm area, digital access, and the Manabí territorial indicator (Table 3).

Compared with holdings whose producer had primary education, holdings whose producer had no formal education had lower adjusted odds of reporting participation in the official FMD vaccination campaign (aOR = 0.52; 95% CI: 0.41–0.67; *p* < 0.001). Higher education was associated with an estimated aOR of 1.49, but its confidence interval included the null value and the *p*-value was 0.051. Secondary education was not associated with the outcome after adjustment.

Compared with dual-purpose holdings, milk-oriented holdings had higher adjusted odds of reporting participation in the official campaign (aOR = 1.37; 95% CI: 1.09–1.74; *p* = 0.008). The estimates for meat-oriented, mixed, and not-classified holdings had confidence intervals that included 1.00.

Current herd size was positively associated with reported participation. A one-unit increase in log(1 + current herd size) was associated with approximately twice the adjusted odds of participation (aOR = 2.11; 95% CI: 1.82–2.46; *p* < 0.001). Total farm area showed an inverse adjusted association: each one-unit increase in log(1 + farm area in hectares) was associated with lower odds of reported participation (aOR = 0.70; 95% CI: 0.62–0.80; *p* < 0.001). These estimates refer to changes on the transformed logarithmic scales and should not be interpreted per additional animal or hectare.

Each additional point in the digital-access score was associated with lower adjusted odds of reporting participation in the official campaign (aOR = 0.85; 95% CI: 0.77–0.94; *p* = 0.002). This association concerns the availability of digital resources and does not demonstrate that digital access itself reduced campaign participation.

After adjustment for geographical region and the remaining covariates, holdings in Manabí had higher adjusted odds of reporting participation than holdings in the other Coast provinces (aOR = 2.87; 95% CI: 1.57–5.26; *p* < 0.001). The Manabí estimate should not be interpreted as a comparison with all other provinces of Ecuador because the model simultaneously included the continental regional classification.

Producer age, producer sex, residence on the holding, affiliation with the Rural Social Insurance Scheme, and the Amazon and Highlands regional contrasts had confidence intervals that included the null value under the principal variance specification. The direction and statistical evidence of the associations with no formal education, milk-oriented production, current herd size, farm area, digital access, and Manabí remained consistent under the alternative singleton-stratum variance specification.

Table 3 presents the complete adjusted estimates for reported participation in the official FMD vaccination campaign.

### 3.4. Factors Associated with Other Cattle Vaccination and Disease-Control Practices

The survey-weighted multivariable model for the reported application of a cattle vaccine other than the official FMD vaccine included 10,538 holdings with complete information. Adjusted associations were observed for current herd size, total farm area, geographical region, and the Manabí territorial indicator (Table 4).

Current herd size was positively associated with reported application of another cattle vaccine. A one-unit increase in log(1 + current herd size) was associated with higher adjusted odds of this outcome (aOR = 1.60; 95% CI: 1.44–1.77; *p* < 0.001). In contrast, a one-unit increase in log(1 + farm area in hectares) was associated with lower adjusted odds (aOR = 0.86; 95% CI: 0.79–0.94; *p* = 0.001). These estimates refer to the transformed logarithmic scales and should not be interpreted per additional animal or hectare.

Marked regional differences were observed. Compared with holdings in the Coast region, holdings in the Amazon had lower adjusted odds of reporting application of another cattle vaccine (aOR = 0.34; 95% CI: 0.23–0.50; *p* < 0.001), as did holdings in the Highlands (aOR = 0.46; 95% CI: 0.33–0.64; *p* < 0.001). After adjustment for geographical region and the remaining covariates, holdings in Manabí had higher adjusted odds than holdings in the other Coast provinces (aOR = 2.33; 95% CI: 1.57–3.45; *p* < 0.001).

The estimate for no formal education was close to the null threshold under the principal variance specification (aOR = 0.83; 95% CI: 0.69–1.00; *p* = 0.053) and was not considered evidence of an adjusted association. Producer age, producer sex, the remaining education categories, cattle-production orientation, digital-access score, residence on the holding, and affiliation with the Rural Social Insurance Scheme had confidence intervals that included 1.00.

The survey-weighted multivariable model for reported implementation of disease-control activities included 10,526 holdings with complete information. Holdings whose producer had no formal education had lower adjusted odds of reporting disease-control activities than holdings whose producer had primary education (aOR = 0.76; 95% CI: 0.62–0.92; *p* = 0.005). Higher and secondary education were not associated with this outcome after adjustment.

Current herd size was positively associated with disease-control activities. A one-unit increase in log(1 + current herd size) was associated with higher adjusted odds of the outcome (aOR = 1.60; 95% CI: 1.44–1.79; *p* < 0.001). Total farm area was not associated with the outcome under the principal variance specification (aOR = 0.92; 95% CI: 0.84–1.01; *p* = 0.083).

Compared with holdings in the Coast, holdings in the Amazon had lower adjusted odds of reporting disease-control activities (aOR = 0.50; 95% CI: 0.33–0.75; *p* = 0.001), as did holdings in the Highlands (aOR = 0.59; 95% CI: 0.42–0.83; *p* = 0.002). Holdings in Manabí had higher adjusted odds of reporting disease-control activities than holdings in the other Coast provinces (aOR = 2.90; 95% CI: 1.86–4.51; *p* < 0.001).

Cattle-production orientation, producer age, producer sex, digital-access score, residence on the holding, and affiliation with the Rural Social Insurance Scheme had confidence intervals that included 1.00 in the disease-control model. All adjusted associations identified for the two outcomes remained consistent in direction and statistical evidence under the alternative singleton-stratum variance specification.

Table 4 presents the complete adjusted estimates for reported application of another cattle vaccine and implementation of disease-control activities.

**Figure 4:**
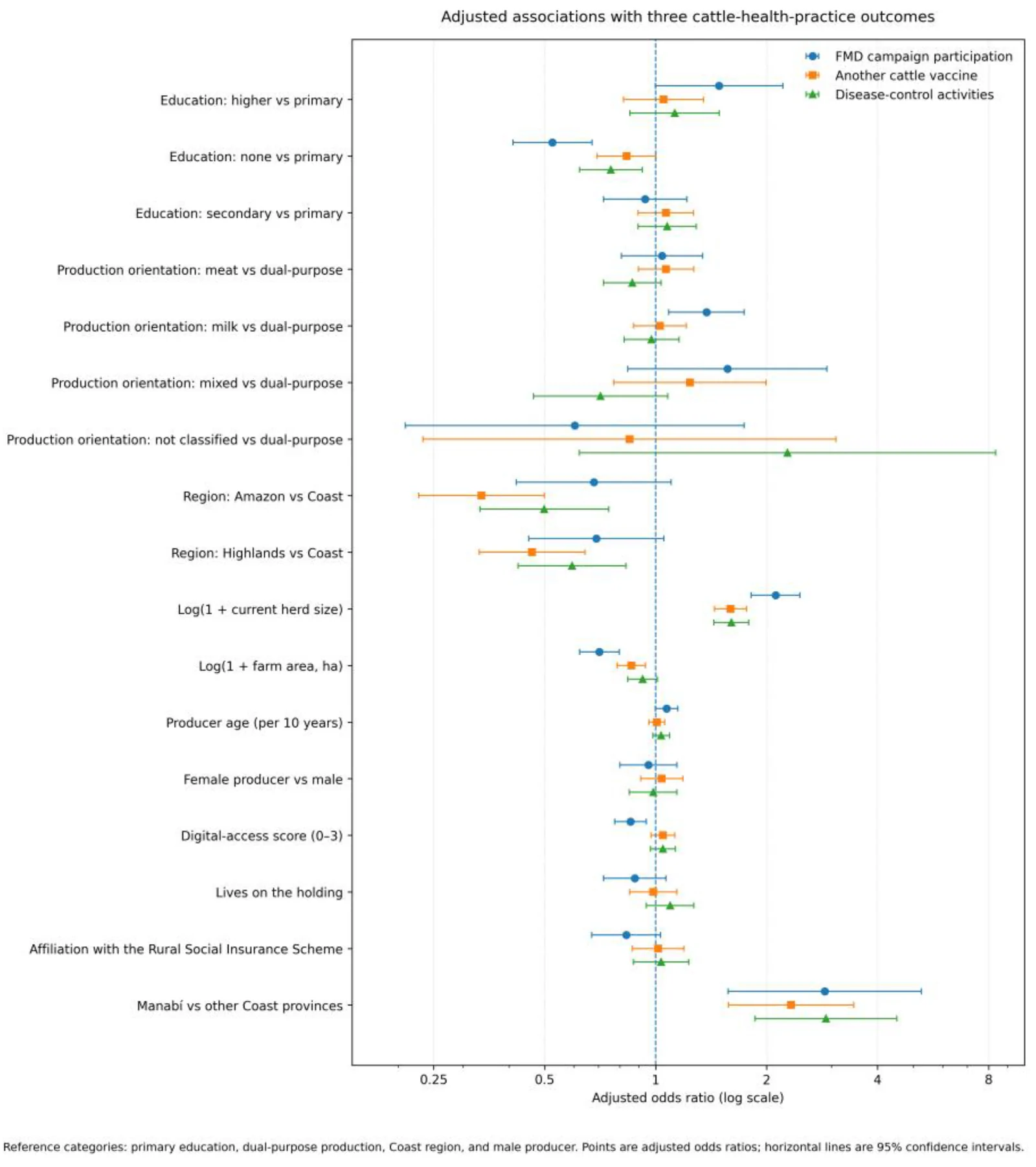
Adjusted associations with the three cattle-health-practice outcomes in the principal continental domain. Note: Points represent adjusted odds ratios, and horizontal lines represent 95% confidence intervals under the principal singleton-stratum variance specification. The vertical dashed line indicates an adjusted odds ratio of 1. Reference categories were primary education, dual-purpose cattle production, the Coast region, and male producer. Current herd size and farm area were modelled as log(1 + x), and producer age was modelled per 10-year increase. The complete-case sample comprised 10,538 holdings for official FMD campaign participation and application of another cattle vaccine and 10,526 holdings for implementation of disease-control activities. Manabí was compared with holdings in the other Coast provinces. FMD, foot-and-mouth disease.

The adjusted associations across the three cattle-health-practice outcomes are summarised in Figure 4.

### 3.5. Factors Associated with Any Self-Reported Principal Cattle Disease

Before dichotomisation, the design-adjusted distribution of the original principal-disease responses was 3.63% for mastitis (95% CI: 3.11–4.15%), 3.55% for other disease (95% CI: 2.99–4.12%), 3.04% for milk fever (95% CI: 2.54–3.54%), 0.47% for vesicular diseases (95% CI: 0.27–0.67%), 0.36% for tympanism (95% CI: 0.18–0.54%), and 0.17% for brucellosis (95% CI: 0.08–0.26%); 88.78% of holdings reported no principal cattle disease (95% CI: 87.81–89.75%). These category-specific estimates are descriptive and should not be interpreted as clinically confirmed disease prevalence.

The survey-weighted multivariable model for any self-reported principal cattle disease included 10,538 holdings with complete information for the outcome and all prespecified covariates. Adjusted associations were observed for cattle-production orientation, geographical region, current herd size, producer education and age, digital access, residence on the holding, and affiliation with the Rural Social Insurance Scheme (Table 5).

Current herd size was positively associated with disease reporting. A one-unit increase in log(1 + current herd size) was associated with higher adjusted odds of reporting a principal cattle disease (aOR = 1.81; 95% CI: 1.58–2.09; *p* < 0.001). Total farm area was not associated with the outcome after adjustment (aOR = 1.06; 95% CI: 0.94–1.18; *p* = 0.359). The herd-size estimate refers to a one-unit change on the transformed logarithmic scale and should not be interpreted as the effect of one additional animal.

Compared with holdings in the Coast region, holdings in the Amazon had higher adjusted odds of reporting a principal cattle disease (aOR = 2.31; 95% CI: 1.47–3.63; *p* < 0.001), as did holdings in the Highlands (aOR = 2.70; 95% CI: 1.86–3.91; *p* < 0.001). After adjustment for geographical region and the remaining covariates, holdings in Manabí did not differ from holdings in the other Coast provinces (aOR = 0.85; 95% CI: 0.52–1.37; *p* = 0.493).

Cattle-production orientation was also associated with disease reporting. Compared with dual-purpose holdings, meat-oriented holdings had lower adjusted odds of reporting a principal cattle disease (aOR = 0.60; 95% CI: 0.47–0.77; *p* < 0.001). Milk-oriented holdings had higher adjusted odds under the principal variance specification (aOR = 1.31; 95% CI: 1.06–1.61; *p* = 0.011), whereas mixed and not-classified holdings had confidence intervals that included 1.00.

Holdings whose producer had no formal education had higher adjusted odds of disease reporting than holdings whose producer had primary education under the principal variance specification (aOR = 1.36; 95% CI: 1.04–1.76; *p* = 0.022). Higher and secondary education were not associated with the outcome. Each additional 10 years of producer age was associated with lower adjusted odds of disease reporting under the principal variance specification (aOR = 0.93; 95% CI: 0.87–0.99; *p* = 0.027). Producer sex was not associated with the outcome.

Each additional point in the digital-access score was associated with higher adjusted odds of reporting a principal cattle disease (aOR = 1.13; 95% CI: 1.04–1.24; *p* = 0.005). Higher adjusted odds were also observed among producers who lived on the holding (aOR = 1.27; 95% CI: 1.07–1.51; *p* = 0.006) and among producers affiliated with the Rural Social Insurance Scheme (aOR = 1.39; 95% CI: 1.12–1.73; *p* = 0.003). These associations concern self-reported disease and may reflect differences in disease occurrence, recognition, observation, access to services, or reporting capacity.

Under the alternative singleton-stratum variance specification, the associations with meat-oriented production, Amazon and Highlands locations, current herd size, digital access, residence on the holding, and affiliation with the Rural Social Insurance Scheme remained statistically supported. The associations with no formal education, milk-oriented production, and producer age retained the same direction but their confidence intervals included 1.00 under the alternative specification. These three results were therefore classified as variance-sensitive rather than fully design-robust. The 95% confidence intervals obtained under the principal and alternative singleton-stratum variance specifications are compared in Supplementary Figure S1.

At the national descriptive level, the alternative singleton-stratum specification increased standard errors by approximately 31% relative to the principal specification (official FMD campaign: 0.655 to 0.858 percentage points; other vaccination: 1.002 to 1.312; disease-control activities: 0.970 to 1.270; self-reported disease: 0.496 to 0.650). Regression point estimates were unchanged because the singleton-stratum procedure affected only variance estimation. The principal conclusions remained stable for the most consistent associations, although selected self-reported-disease coefficients became non-significant under the alternative specification.

Table 5 presents the complete adjusted estimates.

**Figure 5:**
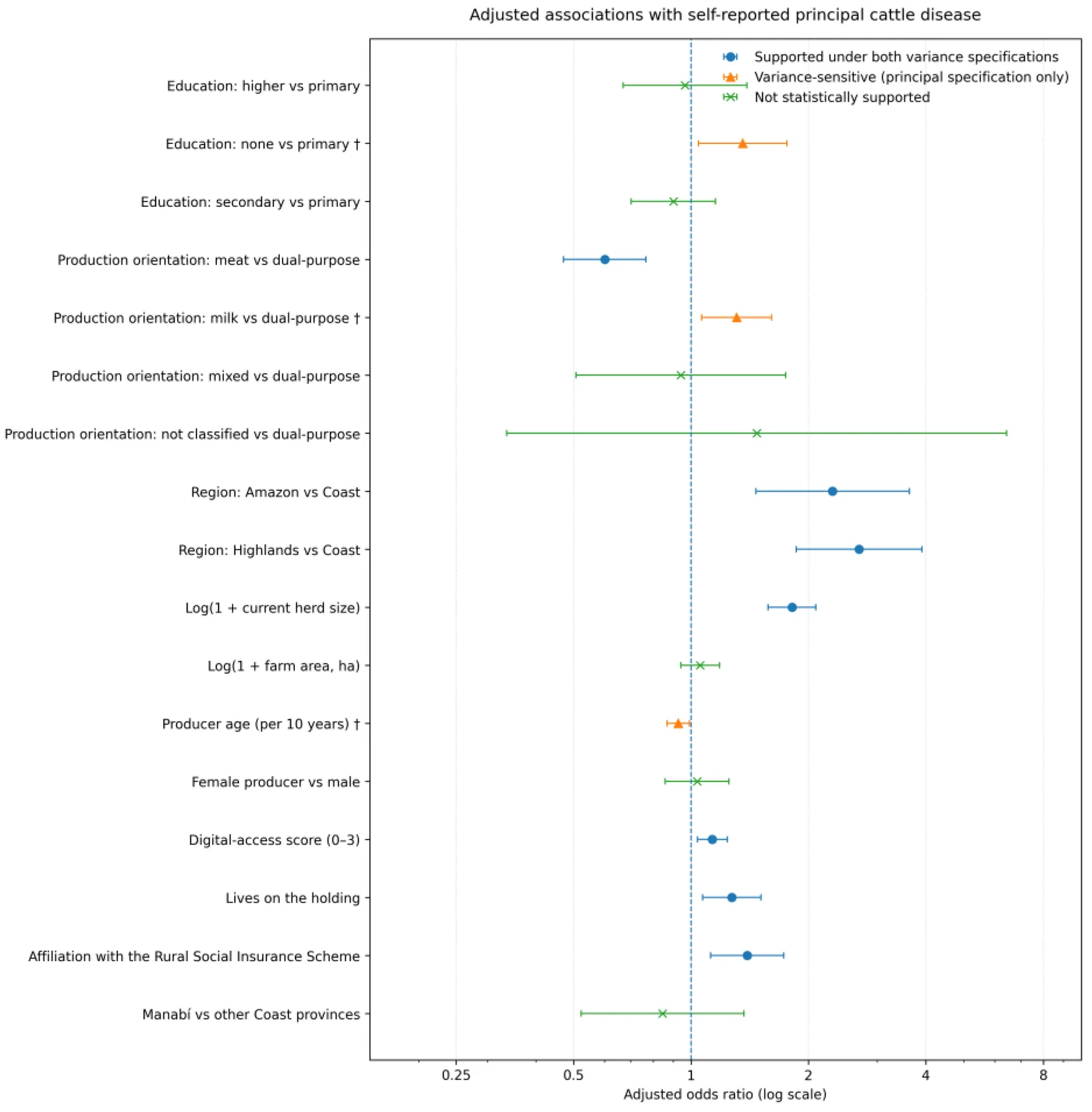
Adjusted associations with self-reported principal cattle disease in the principal continental domain. Note: Points represent adjusted odds ratios, and horizontal lines represent 95% confidence intervals under the principal singleton-stratum variance specification. The vertical dashed line indicates an adjusted odds ratio of 1. Reference categories were primary education, dual-purpose cattle production, the Coast region, and male producer. Current herd size and farm area were modelled as log(1 + x), and producer age was modelled per 10-year increase. The complete-case sample comprised 10,538 holdings. Manabí was compared with holdings in the other Coast provinces. † Association statistically supported under the principal variance specification but not under the alternative singleton-stratum variance specification.

Figure 5 summarises the adjusted associations with self-reported principal cattle disease and distinguishes associations that remained statistically supported under both singleton-stratum variance specifications from those that were variance-sensitive.

## 4. Discussion

The findings are discussed in relation to the organisation of the official FMD vaccination campaign, the implementation of other preventive cattle-health practices, territorial and production-system differences, and the interpretation of self-reported disease. Particular attention is given to the distinction between holding-level participation and animal-level coverage, between preventive engagement and disease reporting, and between adjusted associations and causal effects. The final subsections consider the methodological strengths and limitations of the study and its implications for veterinary policy, surveillance, and future research.

### 4.1. Principal Findings and Overall Interpretation

This study provides survey-design-adjusted evidence on reported cattle-health practices and self-reported disease within the principal continental domain of Ecuador, with a prespecified territorial focus on Manabí. Four main findings emerged. First, reported participation in the official FMD vaccination campaign was more frequent than the application of another cattle vaccine or the implementation of disease-control activities. Second, current herd size was the only farm-level characteristic consistently associated with all four outcomes. Third, substantial territorial differences remained after adjustment for the prespecified farm, producer, and production-system covariates. Fourth, the pattern of factors associated with preventive participation differed from the pattern associated with self-reported disease.

The estimated participation of cattle holdings in the official FMD campaign was 86.38%, compared with 55.96% for the application of another cattle vaccine and 64.74% for disease-control activities. This hierarchy suggests that participation was greatest for the centrally organised national programme. However, the official-campaign result represents holding-level participation and not the proportion of individual cattle vaccinated. It therefore cannot be interpreted as animal-level vaccine coverage, complete herd immunisation, compliance with a vaccination schedule, or evidence of effective immunological protection.

Current herd size was positively associated with each preventive outcome and with self-reported disease. For the preventive outcomes, this pattern may reflect stronger incentives to protect economically valuable herds, greater visibility to veterinary and campaign services, or more formalised herd-health management. For the disease outcome, the interpretation is different: larger herds contain more animals in which a health event may occur and may also have more systematic observation, record keeping, or contact with animal-health personnel. The association with disease reporting therefore does not establish that increasing herd size directly increases individual-animal disease risk.

The territorial results showed a consistent distinction between preventive participation and disease reporting. Manabí had higher descriptive estimates for all three preventive outcomes than the principal continental domain. In the adjusted models, holdings in Manabí also had higher odds of reporting each preventive practice than holdings in the other Coast provinces. By contrast, the Manabí indicator was not associated with self-reported disease after adjustment. This pattern is compatible with comparatively strong preventive participation in Manabí, but it should not be interpreted as an isolated causal effect of province. The Manabí indicator may capture differences in programme delivery, veterinary-service organisation, production systems, market integration, accessibility, or producer networks that were not measured directly.

A different territorial pattern was observed for the broader regional contrasts. Compared with holdings in the Coast, holdings in the Amazon and Highlands had lower adjusted odds of reporting another cattle vaccine and disease-control activities but higher adjusted odds of self-reported disease. Region is a composite contextual variable and may represent differences in production systems, climate, herd composition, accessibility, veterinary-service availability, disease ecology, animal movement, and reporting behaviour. The regional coefficients should consequently be interpreted as adjusted territorial associations requiring further epidemiological investigation rather than as direct effects of geographical residence.

Production orientation and producer characteristics were not associated uniformly across the four outcomes. Milk-oriented production was associated with higher participation in the official FMD campaign, whereas its positive association with disease reporting was sensitive to the singleton-stratum variance specification. Meat-oriented holdings had lower adjusted odds of self-reported disease, and this association remained supported under both variance procedures. Producers with no formal education had lower adjusted odds of official-campaign participation and disease-control activities; their higher adjusted odds of disease reporting under the principal variance specification were not retained under the alternative specification. These differences indicate that findings supported by only one variance specification require more cautious interpretation than the design-robust associations.

Digital access was inversely associated with reported participation in the official FMD campaign but positively associated with self-reported disease. These apparently contrasting findings should not be interpreted as evidence that digital resources reduce vaccination or cause disease. The digital-access score may capture differences in farm organisation, information access, territorial connectivity, service use, disease recognition, or willingness to report. Similarly, residence on the holding and affiliation with the Rural Social Insurance Scheme were positively associated with self-reported disease but not with the preventive outcomes, potentially reflecting greater opportunity to observe animals or stronger contact with institutional services.

Taken together, the findings show that preventive participation and self-reported disease represent distinct dimensions of cattle health. A holding may report vaccination and disease-control activities while also reporting disease, particularly when it has a larger herd, more frequent animal observation, or stronger contact with veterinary or institutional services. Because the study is observational and cross-sectional, the results represent adjusted associations among reported practices, holding characteristics, territorial context, and disease reporting. They do not estimate vaccine effectiveness, establish temporal direction, or demonstrate causal effects on disease occurrence.

### 4.2. Participation in the Official Foot-and-Mouth Disease Vaccination Campaign

Reported participation in the official FMD vaccination campaign was the most frequent preventive outcome examined in this study. Within the principal continental domain, 86.38% of cattle holdings reported participation. This high proportion is consistent with the scale of the national programme, but it should be interpreted as the result of interacting organisational and operational processes rather than central coordination alone. At the organisational level, central coordination can structure vaccine supply, campaign timing, communication, and institutional oversight. At the operational level, actual holding-level participation depends on territorial implementation, access to cattle holdings, availability of personnel, animal handling, and producer cooperation. The ESPAC data do not allow these two levels to be separated empirically, and the observed participation estimate therefore reflects their combined operation. The campaign conducted from 14 February to 31 March 2025 was intended to immunise more than 4.7 million cattle and buffaloes, while official information subsequently reported that more than 4 million animals were immunised during the final vaccination phase conducted later in 2025 [6,7].

Because ESPAC records holding-level participation rather than animal-level vaccination status, the 86.38% estimate should be interpreted as programme reach among holdings, not as the proportion of cattle vaccinated or as evidence of complete herd immunisation or immunological protection.

The adjusted results indicate that participation was not distributed uniformly across producer and holding characteristics. Holdings whose producer had no formal education had approximately half the adjusted odds of reporting participation compared with holdings whose producer had primary education. This association remained supported under both singleton-stratum variance specifications. Although ESPAC does not measure knowledge of FMD, understanding of vaccination requirements, trust in Veterinary Services, or accessibility of campaign information, previous studies have identified disease knowledge, communication, distance, affordability, service availability, and operational constraints as relevant determinants of livestock-vaccination participation [11,13,20,21].

Formal education should not be interpreted as a direct determinant of campaign compliance. Educational attainment may represent differences in access to information, interaction with veterinary personnel, ability to navigate administrative procedures, participation in producer organisations, or broader socioeconomic and geographical conditions. The practical implication is that campaign communication and producer engagement should remain understandable and accessible to cattle keepers with different educational backgrounds rather than relying exclusively on written or digitally mediated information.

Milk-oriented holdings had higher adjusted odds of campaign participation than dual-purpose holdings. Dairy systems may involve more frequent animal handling and more regular interaction with veterinarians, milk buyers, producer organisations, or public animal-health services, potentially facilitating campaign notification and access to cattle. However, ESPAC does not measure production intensity, milk-marketing arrangements, association membership, or the frequency of veterinary contact. The result should therefore be interpreted as an adjusted production-system association rather than as evidence that milk orientation itself causes greater participation.

Current herd size was the strongest continuous correlate of official-campaign participation. Larger herds may represent greater economic value at risk, stronger incentives to avoid production losses, greater visibility to Veterinary Services, or more frequent interaction with animal-health and livestock-movement systems. Conversely, larger farm area was associated with lower adjusted odds after herd size and the remaining covariates were considered. Herd size and land area therefore represent different dimensions of production scale. For a comparable transformed herd size, a larger area may correspond to a more extensive or spatially dispersed system, although accessibility, cattle distribution, handling infrastructure, and vaccination logistics were not measured directly [13,21].

The inverse association between the digital-access score and campaign participation also requires cautious interpretation. The score measured access to selected digital resources but did not indicate whether producers received animal-health messages, used AGROCALIDAD platforms, registered for the campaign electronically, or possessed digital veterinary literacy. The result does not demonstrate that digital access reduced vaccination behaviour. It may instead reflect residual differences in production systems, producer profiles, territorial connectivity, or the channels through which the campaign reached cattle holdings.

Manabí retained higher adjusted odds of reported campaign participation than the other Coast provinces. The Amazon and Highlands regional contrasts did not differ statistically from the Coast after adjustment, indicating that the Manabí result should not be generalised as an overall Coast advantage. The province-level association may reflect local campaign organisation, the economic importance of cattle production, producer mobilisation, livestock networks, accessibility, or institutional coverage. Because these mechanisms were not measured by ESPAC, the coefficient represents a robust adjusted territorial association and not an intrinsic or causal effect of being located in Manabí.

The timing of ESPAC 2025 gives these findings particular policy relevance. Ecuador officially notified the World Organisation for Animal Health of the cessation of routine FMD vaccination in cattle and buffaloes from 29 January 2026 as part of its planned progression towards recognition as free from FMD without vaccination [7]. Nevertheless, under the official status adopted by WOAH in May 2026, continental Ecuador remained recognised as an FMD-free zone where vaccination was practised, while Galápagos remained an FMD-free zone where vaccination was not practised [3]. The cessation of vaccination and the subsequent international recognition of a different official status are therefore distinct processes.

The ESPAC estimates consequently provide a holding-level baseline from the final year of routine vaccination. They do not constitute evidence either supporting or opposing the subsequent policy change. Their value lies in identifying producer groups, production settings, and territories in which reported participation was comparatively lower before the transition. During the post-routine-vaccination period, effective producer contact, early recognition and notification of suspected vesicular disease, diagnostic capacity, traceability, movement control, biosecurity, and emergency preparedness become increasingly important. The national FMD simulation conducted in May 2026 illustrates the operational emphasis placed on surveillance, timely detection, animal movement, institutional coordination, and emergency response [22]. Holdings that were less connected to the vaccination programme may require targeted engagement to ensure their effective participation in this surveillance-centred strategy.

### 4.3. Other Cattle Vaccination and Disease-Control Practices

Reported application of another cattle vaccine and implementation of disease-control activities were less frequent than participation in the official FMD vaccination campaign. Within the principal continental domain, 55.96% of cattle holdings reported applying another vaccine, whereas 64.74% reported implementing disease-control activities. This difference indicates that engagement outside the centrally organised FMD campaign was less uniform. However, ESPAC does not establish whether these activities were initiated preventively, implemented in response to a previous or suspected health problem, recommended by a veterinarian, or required through another animal-health programme.

The two outcomes represent broad and analytically distinct constructs. The vaccination indicator establishes only whether a vaccine other than the official FMD vaccine was reportedly applied. It does not identify the targeted disease, vaccine product, number or proportion of animals vaccinated, timing, protocol completeness, booster administration, or cold-chain conditions. Similarly, the disease-control indicator records whether an activity was reported but does not identify the specific measure, its frequency, technical quality, intensity, or pathogen-specific suitability. The estimates should therefore be interpreted as holding-level reports of vaccination and disease-control activity rather than comprehensive assessments of herd-health programme quality. Accordingly, higher reported participation cannot be interpreted as evidence of higher-quality, more complete, more appropriate, or more effective vaccination or disease-control implementation.

Current herd size was positively associated with both outcomes. A one-unit increase in log(1 + current herd size) was associated with an aOR of approximately 1.60 for both application of another cattle vaccine and implementation of disease-control activities. Larger herds may involve greater economic value at risk, more formalised management, stronger incentives to limit production losses, or more frequent contact with veterinary services. Evidence from other livestock settings has similarly identified herd scale, disease knowledge, service availability, previous disease experience, and access to veterinary personnel as factors associated with vaccine use and veterinary-service utilisation [13,21,23]. Nevertheless, the binary ESPAC outcomes do not establish that larger holdings implemented technically more complete or higher-quality programmes. The parallel positive association between herd size and self-reported disease requires a different interpretation. Larger operations may have more systematic disease monitoring, record keeping, and veterinary contact, increasing the probability that health events are recognised and reported. Consequently, the higher probability of disease reporting among larger holdings does not necessarily imply a higher underlying incidence of disease.

Farm area showed a different pattern from herd size. After adjustment for herd size and the remaining covariates, larger farm area was associated with lower adjusted odds of applying another cattle vaccine, whereas its association with disease-control activities was not statistically supported. Herd size and land area should therefore not be treated as interchangeable indicators of production scale. For a comparable transformed herd size, a larger area may represent a more extensive or spatially dispersed production system involving greater travel distances, more difficult animal gathering, or higher logistical costs. These mechanisms were not measured directly, but previous studies have identified distance, vaccine availability, affordability, service quality, and operational accessibility as barriers to veterinary-vaccine use [13,21].

Formal education was associated specifically with the disease-control outcome. Holdings whose producer had no formal education had lower adjusted odds of reporting disease-control activities than holdings whose producer had primary education. For application of another cattle vaccine, the estimate for no formal education was in the same direction, but its confidence interval included 1.00 and the *p*-value was 0.053; it should therefore not be presented as evidence of an adjusted association. Educational attainment may represent differences in access to technical information, communication with veterinarians, familiarity with administrative processes, participation in producer organisations, or capacity to translate general animal-health recommendations into routine holding-level activities. Research on veterinarian–farmer relationships also indicates that trust, collaborative communication, time, and perceived feasibility influence the implementation of farm biosecurity and disease-control recommendations [24].

Marked territorial differences remained after adjustment. Compared with holdings in the Coast, holdings in the Amazon and Highlands had lower adjusted odds of both applying another cattle vaccine and implementing disease-control activities. These regional associations may represent differences in cattle-production systems, territorial accessibility, veterinary-service availability, animal movement, disease priorities, institutional programme reach, or reporting behaviour. The available ESPAC variables do not permit these possible mechanisms to be separated, and the regional coefficients should not be interpreted as direct effects of geographical residence.

These regional contrasts are broadly consistent with evidence from small-scale livestock systems in Ethiopia, Sri Lanka, Myanmar, and Ghana, where vaccination participation has been associated with production context, service accessibility, vaccine and vaccinator availability, and access to information [9,11,12,13]. In Ecuador, the Coast–Highlands–Amazon pattern may therefore reflect interacting territorial differences in service delivery and production conditions rather than geographical location itself. Because these mechanisms were not measured directly in ESPAC, this comparison should be regarded as contextual rather than causal.

Manabí showed a distinct pattern within the Coast. Holdings in Manabí had higher adjusted odds of applying another cattle vaccine and implementing disease-control activities than holdings in the other Coast provinces. The simultaneous presence of regional and province-specific associations indicates that the findings cannot be reduced to a general Coast advantage. The Manabí indicator may capture differences in livestock-sector organisation, veterinary-service delivery, producer networks, commercial integration, institutional activity, or the composition of cattle-production systems. Because these characteristics were not measured directly, the coefficient should be interpreted as an adjusted territorial marker requiring further investigation rather than as evidence that location in Manabí causes greater engagement in vaccination or disease-control activities.

Producer age, producer sex, cattle-production orientation, digital-access score, residence on the holding, and affiliation with the Rural Social Insurance Scheme were not associated with either outcome after adjustment. These findings do not demonstrate that the corresponding characteristics are irrelevant to cattle-health management. Their relationships may operate indirectly through herd scale, education, territorial accessibility, veterinary contact, or socioeconomic conditions not represented in the models. In particular, the digital-access score measured general access to selected technologies rather than the use of digital veterinary services or receipt of animal-health information.

Overall, the findings support a differentiated approach to the delivery of vaccination and disease-control services. Holdings with smaller herds, producers with limited formal education, and territories with lower reported engagement may require more accessible communication, locally adapted technical support, mobile or decentralised veterinary provision, and stronger links with producer organisations. Such strategies should address not only technical information but also vaccine availability, transport, animal handling, affordability, trust, continuity of veterinary support, and the practical feasibility of implementing recommended measures. Vaccination should form part of a broader herd-health approach that also includes biosecurity, management of animal introductions and movements, cleaning and disinfection, surveillance, and prompt reporting of suspected disease.

### 4.4. Self-Reported Principal Cattle Disease and Surveillance Implications

Within the principal continental domain, 11.22% of cattle holdings reported at least one principal cattle disease. This outcome requires a different interpretation from the three cattle-health-practice indicators. It was derived from the condition identified by the producer as the principal disease affecting cattle on the holding and therefore represents producer-reported recognition of a health event rather than clinically assessed morbidity, laboratory-confirmed infection, or animal-level disease prevalence.

The outcome combined milk fever, mastitis, tympanism, brucellosis, vesicular diseases, and other reported conditions. These categories differ in aetiology, transmission, clinical presentation, diagnostic requirements, and control measures. The survey did not establish the number or proportion of affected animals, the date or duration of the event, clinical severity, veterinary confirmation, laboratory testing, treatment, mortality, or formal notification. The estimate should consequently not be interpreted as the prevalence or incidence of a single disease or as a homogeneous measure of cattle morbidity.

Current herd size was the strongest design-robust farm-level correlate of disease reporting. Larger holdings had higher adjusted odds of reporting a principal cattle disease. Even if the individual-animal probability of disease were similar across holdings, a larger herd would provide more opportunities for at least one health event to occur or be observed. Larger operations may also differ in animal movement, management complexity, monitoring, record keeping, veterinary contact, and treatment history. The association may therefore reflect a combination of cumulative population at risk, disease occurrence, observation intensity, and reporting capacity rather than a direct causal effect of herd expansion.

Cattle-production orientation was also relevant, although the results require different levels of caution. Meat-oriented holdings had lower adjusted odds of disease reporting than dual-purpose holdings, and this association remained supported under both variance specifications. The lower odds should not be interpreted as evidence that meat-oriented herds had a lower incidence of every infectious, parasitic, metabolic, or reproductive condition. Differences in animal handling, frequency of observation, production monitoring, and the composition of the outcome may influence which conditions are recognised and reported.

Milk-oriented holdings had higher adjusted odds under the principal variance specification, but this association was not statistically supported under the alternative singleton-stratum specification. It should therefore be regarded as variance-sensitive. The composition of the outcome is particularly relevant because it explicitly included mastitis and milk fever, conditions linked to lactation, udder health, milking procedures, and the physiological demands of milk production. Nevertheless, a general milk-production classification cannot represent the considerable variation in hygiene, housing, equipment, pasture access, lactation management, and veterinary care that influences mastitis occurrence [25]. The result does not establish that milk-oriented systems were intrinsically less healthy.

Holdings in the Amazon and Highlands had higher adjusted odds of reporting a principal cattle disease than holdings in the Coast. These design-robust regional associations may reflect differences in cattle-production systems, environmental exposure, vector ecology, altitude, rainfall, pasture conditions, animal movement, herd composition, veterinary-service availability, or the diseases that producers recognise as important. They may also reflect differences in terminology, veterinary access, diagnostic opportunity, and reporting behaviour. The regional coefficients consequently indicate differences in the adjusted probability of reporting a principal disease and not confirmed differences in pathogen circulation or total disease incidence.

Digital access, residence on the holding, and affiliation with the Rural Social Insurance Scheme were positively associated with disease reporting under both variance specifications. These characteristics are more plausibly interpreted as potential indicators of observation and institutional connection than as direct biological risk factors. Producers living on the holding may observe cattle more frequently, while digital or institutional connectivity may facilitate information seeking, communication with veterinary personnel, or recognition of clinical signs. However, the digital-access score did not measure the use of veterinary platforms, receipt of animal-health information, formal disease reporting, or access to diagnostic services. Affiliation with the Rural Social Insurance Scheme may similarly reflect broader socioeconomic or institutional integration rather than a direct pathway affecting cattle disease.

The associations with no formal education, producer age, and milk-oriented production were supported only under the principal variance specification and were therefore treated as exploratory variance-sensitive findings rather than as robust epidemiological evidence. These findings should not form the basis of strong epidemiological or policy conclusions unless they are confirmed using additional data and alternative analytical designs. By contrast, the associations with herd size, meat-oriented production, Amazon and Highlands locations, digital access, residence on the holding, and affiliation with the Rural Social Insurance Scheme remained supported under both variance specifications.

The Manabí results illustrate the importance of distinguishing descriptive from adjusted territorial comparisons. The design-adjusted proportion reporting disease was lower in Manabí than in the principal continental domain, but the adjusted Manabí coefficient did not differ from that of the other Coast provinces. The descriptive difference may therefore reflect, at least partly, the composition of cattle holdings and producers in Manabí rather than an independent province-level association. The simultaneous finding of greater preventive participation and no adjusted difference in disease reporting does not demonstrate either that preventive practices eliminated disease or that they were ineffective.

Temporal ordering cannot be established from the cross-sectional data. Vaccination or disease-control activities may have preceded a reported health event, may have been implemented in response to an earlier event, or may have targeted a different condition. Holdings with stronger veterinary engagement may also be more likely to recognise and report disease. The regression coefficients must therefore not be interpreted as estimates of vaccine effectiveness or of the causal effects of disease-control activities.

Despite these constraints, producer-reported disease has potential value as an initial surveillance signal. The recognition of unusual clinical events by cattle keepers may precede contact with veterinarians or competent authorities, and effective passive surveillance depends partly on the willingness and ability of producers to report suspected disease. Evidence from cattle-surveillance research indicates that trust in veterinarians, confidence in institutions, and accessible professional relationships can support participation in passive surveillance [26]. Self-reported survey indicators cannot replace clinical investigation, laboratory confirmation, formal notification, or structured active and passive surveillance, but they can help identify territories and categories of holdings in which disease recognition, veterinary access, and reporting pathways warrant further assessment.

Future ESPAC editions could strengthen the epidemiological value of the cattle-health module by recording each disease or clinical syndrome separately, the approximate date of occurrence, the number of affected and dead animals, whether a veterinarian was consulted, whether laboratory testing was performed, the measures adopted, and whether the event was formally notified. These additions would allow clearer differentiation among disease occurrence, producer recognition, veterinary diagnosis, treatment, control, mortality, and official notification. The present findings should therefore be interpreted primarily as evidence of variation in reported cattle-health events according to herd structure, production orientation, territorial context, observation, and institutional connection rather than as estimates of pathogen-specific disease burden.

### 4.5. Methodological Strengths and Limitations

A principal strength of this study is the use of nationally distributed public-use microdata from an official agricultural survey. The initial cattle-module database contained 10,715 uniquely identified records, all of which were successfully linked to the corresponding producer-characteristics records. Design-based estimation incorporated the final ESPAC expansion weight, sampling strata, and PSUs, thereby accounting for unequal selection probabilities, stratification, and within-PSU dependence rather than treating the observations as a simple random sample.

A second strength is the explicit distinction between holding-level survey indicators and animal-level epidemiological measures. The reported numbers of vaccinated or affected cattle were not divided by current herd size because the numerator and denominator referred to different temporal points. The outcomes were instead operationalised as binary holding-level indicators: participation in the official FMD vaccination campaign, application of another cattle vaccine, implementation of disease-control activities, and reporting of any principal cattle disease. This decision avoided presenting mathematically calculable but epidemiologically invalid estimates of vaccination coverage or disease prevalence.

The analytical design also distinguished broad regional patterns from the prespecified province-level question concerning Manabí. Geographical region and the Manabí indicator were included simultaneously, allowing the Manabí coefficient to be interpreted as the adjusted contrast with holdings in the other Coast provinces. Santa Elena was retained for descriptive presentation but was not represented by a separate regression coefficient because its sample was small and showed no observed variation in the three cattle-health-practice outcomes. This prevented unstable or unidentified estimates from being interpreted as substantive province-level evidence.

Additional strengths include the use of a common prespecified covariate structure across the four models, explicit reporting of outcome-specific complete-case sample sizes, and sensitivity analysis of the singleton-stratum variance procedure. The alternative variance specification preserved the point estimates but modified their estimated precision, allowing associations that depended on the treatment of singleton strata to be identified as variance-sensitive. Data-processing decisions, exclusions, derived variables, model outputs, and sensitivity procedures were retained in a reproducibility package to support verification of the analytical workflow.

The study nevertheless has several limitations. First, its cross-sectional design does not establish the temporal order among cattle-health practices, holding characteristics, and reported disease. Vaccination or disease-control activities may have preceded a health event, may have been implemented in response to an earlier event, or may have targeted a condition different from the one subsequently reported. The regression coefficients therefore describe adjusted associations and cannot be interpreted as causal effects, risk ratios, or estimates of vaccine or intervention effectiveness.

Second, all four outcomes were based on producer responses rather than independent veterinary verification. Reporting may have been influenced by recall, interpretation of the questionnaire, local terminology, veterinary access, disease awareness, and willingness to disclose practices or health events. The official FMD indicator did not establish the number or proportion of eligible animals vaccinated, protocol completeness, timing, or immunological protection. The other-vaccination and disease-control indicators did not identify the specific vaccine, targeted condition, intervention, frequency, intensity, or technical quality.

The principal-disease outcome presents additional measurement constraints because it combines infectious, metabolic, digestive, mammary, vesicular, and other reported conditions within one binary indicator. These conditions have different aetiologies, diagnostic requirements, transmission pathways, and control strategies. The survey did not provide the date or duration of the event, the number of affected or dead animals, clinical severity, veterinary confirmation, laboratory testing, treatment, or formal notification. The outcome is therefore suitable for examining patterns of disease reporting but not for estimating pathogen-specific prevalence, incidence, mortality, or disease burden.

Residual confounding is also likely. The public-use data did not include detailed measures of veterinary-service availability, travel time to service points, producer income, cattle density, breed composition, animal purchases and movements, pasture management, housing, milking hygiene, biosecurity infrastructure, previous outbreaks, producer-organisation membership, commercial integration, or trust in animal-health institutions. The regional and Manabí coefficients may consequently summarise several unmeasured institutional, environmental, economic, and production-system characteristics.

The models used a common adjustment set to facilitate comparison across outcomes. Herd size and farm area were log-transformed to reduce skewness, whereas the other continuous predictors were entered linearly on the logit scale. This specification may not capture thresholds, nonlinear relationships, or interactions among herd scale, production orientation, education, and territorial context. The models should therefore be regarded as parsimonious associational models rather than exhaustive representations of cattle-health behaviour or disease occurrence.

No statistical imputation was performed, and each model used an outcome-specific complete-case sample. Although the resulting samples remained large—10,538 holdings for the official FMD vaccination, other-vaccination, and disease models and 10,526 for the disease-control model—complete-case analysis can introduce selection bias when missingness is related to unobserved characteristics or to the outcome after conditioning on the included covariates. The limited amount of missing information reduces but does not eliminate this possibility.

Because four related models evaluated multiple coefficients, the analysis involved a substantial number of hypothesis tests, increasing the possibility of chance findings. No formal correction for multiple comparisons was applied. Accordingly, individual *p*-values—particularly those close to 0.05—were interpreted cautiously and alongside effect magnitude, confidence-interval width, consistency across related outcomes, epidemiological plausibility, and stability under the alternative variance specification. Associations supported only under the principal variance procedure were explicitly identified as variance-sensitive and require confirmation in independent data.

Variance estimation was complicated by the presence of 58 strata represented by a single observed PSU in the complete positive-weight design. The principal and alternative singleton-stratum specifications produced identical point estimates but different standard errors, confidence intervals, and *p*-values. The principal conclusions were retained for the most consistent associations, including current herd size, several regional contrasts, and the Manabí associations with the three cattle-health-practice outcomes. Nevertheless, the sensitivity of selected disease-model coefficients demonstrates that inferential conclusions can depend on the variance specification used for sparse survey strata.

The geographical scope of the principal analysis was restricted to positive-weight holdings assigned to one of the 23 provinces of continental Ecuador. Records assigned to the official “undelimited zone” category were excluded from territorial analyses, and Galápagos was outside the geographical scope of ESPAC 2025. The results should therefore not be generalised to Galápagos or to records that could not be assigned consistently to the predefined territorial classification. Santa Elena requires particular caution. The 26 records were not the result of an author-defined subsampling decision; they corresponded to all cattle-module records from Santa Elena available in the ESPAC 2025 public-use dataset, and all 26 had a positive final expansion weight. Because the realised provincial sample was small and all 26 holdings reported each of the three cattle-health-practice outcomes, the separate Santa Elena estimates contained no observed within-domain variation for those outcomes. We therefore retained Santa Elena in the descriptive domain analysis for completeness, with its unweighted sample size explicitly reported, but did not fit a Santa Elena-specific regression coefficient and do not interpret the 100% estimates as precise evidence of complete provincial participation. Santa Elena remained part of the Coast region in the regional models, while its stand-alone provincial estimates should be regarded as descriptive and interpreted cautiously, particularly for self-reported disease, for which the confidence interval was very wide.

Finally, the findings describe reported cattle-health practices and disease recognition during the ESPAC 2025 reference period. They provide a baseline from the final year of routine FMD vaccination but cannot establish how vaccination behaviour, surveillance participation, veterinary-service use, or disease reporting changed after routine vaccination ceased in 2026. Evaluation of that transition will require repeated cross-sectional or longitudinal data collected under the subsequent surveillance-centred context.

### 4.6. Implications for Veterinary Policy, Surveillance, and Future Research

The findings have implications for the organisation of cattle-health services in Ecuador. Reported participation in the official FMD vaccination campaign was substantially more frequent than application of another cattle vaccine or implementation of disease-control activities. This contrast is consistent with broad holding-level reach under a centrally coordinated programme, but it does not demonstrate that cattle producers have continuous access to comprehensive vaccination schedules, veterinary advice, biosecurity planning, diagnostic services, or disease-specific control programmes. Animal-health policy should therefore distinguish participation in a national campaign from sustained engagement in broader herd-health management.

Herd size provides one potential criterion for differentiated service planning. Larger holdings had higher adjusted odds of reporting all three cattle-health-practice outcomes, but they also had higher odds of reporting a principal cattle disease. Larger herds may be comparatively visible to veterinary and institutional services while simultaneously representing more animals at risk, greater management complexity, and potentially greater epidemiological consequences if disease occurs. They therefore continue to warrant risk-based surveillance, traceability, movement oversight, and access to veterinary support.

Smaller cattle holdings present a different service-delivery challenge. Their lower adjusted odds of reporting the three cattle-health practices may reflect weaker contact with veterinary services, less integration into formal livestock networks, logistical barriers, or differences in perceived economic risk. These mechanisms were not measured directly, but the observed pattern supports evaluating mobile or decentralised veterinary provision, locally scheduled activities, community-based communication, and collaboration with producer organisations in settings where cattle holdings are small, dispersed, or weakly connected to institutional channels.

The education results also have practical implications, but they should not be generalised beyond the outcomes in which associations were observed. Producers with no formal education had lower adjusted odds of participating in the official FMD campaign and of reporting disease-control activities. Communication strategies should therefore use accessible language, practical demonstrations, appropriate visual materials, locally familiar terminology, and opportunities for direct dialogue with veterinary personnel. The objective should extend beyond distributing information and should include ensuring that producers understand the purpose, timing, target animals, repetition requirements, and reporting implications of each intervention.

Territorial differences support context-sensitive implementation of national animal-health standards and differentiated operational priorities across the three major regions. In the Coast, where descriptive participation in all three cattle-health practices was comparatively high, policy should prioritise sustaining programme reach, maintaining surveillance sensitivity, and ensuring continued producer contact and rapid notification after the transition away from routine FMD vaccination. In the Highlands, lower reported participation in other vaccination and disease-control activities, together with higher adjusted odds of self-reported disease, supports strengthening routine veterinary outreach, producer communication, and access to herd-health services beyond nationally organised campaigns. In the Amazon, the combination of the lowest descriptive estimate for other vaccination, comparatively low disease-control participation, and higher adjusted odds of self-reported disease indicates a need to evaluate geographical accessibility, mobile or decentralised veterinary provision, diagnostic and notification pathways, and continuity of services for dispersed holdings. National technical standards may remain uniform, but their delivery mechanisms may need to be adapted to territorial conditions. These priorities are suggested by the observed patterns and should not be interpreted as evidence that any specific unmeasured service deficit caused the regional differences.

Manabí represents a potentially informative setting for implementation research. Holdings in the province had higher adjusted odds of reporting all three cattle-health practices than holdings in the other Coast provinces, while the Manabí indicator was not associated with self-reported disease. This pattern does not establish that a specific provincial programme caused the difference. A focused implementation study could examine whether veterinary-service organisation, producer mobilisation, livestock-sector networks, accessibility, commercial integration, or other local characteristics contributed to the observed participation pattern and whether any effective practices are transferable to other territories.

The findings are also relevant to surveillance following the cessation of routine FMD vaccination. In this post-routine-vaccination context, animal-health protection depends increasingly on early recognition, rapid reporting, diagnostic confirmation, traceability, movement control, biosecurity, and emergency preparedness [7,22]. Holdings and producer groups that were comparatively less likely to participate in the previous vaccination programme may require targeted engagement to ensure that they remain connected to surveillance and notification systems.

Digital tools may support communication and surveillance, but the present results do not justify using general technology ownership as a proxy for digital veterinary engagement. The digital-access score was inversely associated with official-campaign participation and was not associated with application of another vaccine or disease-control activities. Future programmes should therefore evaluate the actual use, accessibility, and effectiveness of veterinary communication platforms, mobile reporting systems, electronic animal identification, appointment systems, and producer information services rather than assuming that general access to digital devices improves programme participation.

The self-reported disease findings further indicate that passive surveillance cannot be assessed solely by counting reports. Reporting depends on disease occurrence, producer recognition, frequency of animal observation, access to professional advice, trust in veterinarians and authorities, and the perceived consequences of notification. Surveillance-strengthening initiatives should consequently address both technical capacity and professional relationships. Accessible veterinary contact, clear feedback after notification, transparent investigation procedures, and sustained trust between producers and Veterinary Services may support participation in farm-level biosecurity and passive surveillance [24,26].

Several priorities emerge for future quantitative research. The cessation of routine FMD vaccination in 2026 creates an important opportunity to evaluate changes before and after the policy transition, using ESPAC 2025 as a pre-transition baseline. If subsequent ESPAC editions retain comparable cattle-health variables, repeated cross-sectional analyses could assess changes in preventive practices and disease reporting across the principal continental domain and geographical regions. With several comparable pre- and post-transition observations, interrupted time-series approaches could be considered. Controlled interrupted time-series or difference-in-differences designs would require a defensible comparison population or exposure contrast and verification of the corresponding design assumptions. If future data permit confidential longitudinal linkage of the same holdings, panel-data models could provide stronger evidence on within-holding changes. These approaches would strengthen causal assessment, although interpretation would remain conditional on data comparability, policy timing, and the assumptions of the selected design. Independent replication of the present design-based estimates would also strengthen the evidence base for monitoring change over time.

Future survey instruments should collect more detailed information on vaccination and disease-control activities. Vaccination variables should identify the disease targeted, vaccine provider, date of administration, number and proportion of animals vaccinated, number of doses, booster completion, payment, and reasons for non-participation. Disease-control variables should distinguish among quarantine, control of animal introductions, isolation, cleaning and disinfection, parasite control, vector control, movement restrictions, carcass disposal, diagnostic testing, and veterinary herd-health planning.

Disease information should likewise be disaggregated. Recording each disease or clinical syndrome separately, together with the approximate date of occurrence, number of affected and dead animals, veterinary consultation, laboratory confirmation, treatment, control measures, and formal notification, would permit pathogen- or syndrome-specific analysis. Such information would improve differentiation among disease occurrence, recognition, diagnosis, response, mortality, and official reporting.

Qualitative research would complement the survey findings by examining why producers participate in or remain disconnected from vaccination, disease-control, and surveillance activities. Interviews with cattle producers, private veterinarians, AGROCALIDAD personnel, livestock associations, and local authorities could clarify the roles of trust, cost, accessibility, communication, previous disease experience, service continuity, and perceptions of institutional programmes. These mechanisms cannot be inferred reliably from the present regression coefficients.

Spatial analysis could also be expanded if suitably anonymised geographical information becomes available. Linking holdings or appropriately protected small-area aggregates with veterinary-service locations, road accessibility, cattle density, animal-movement networks, climatic conditions, and officially notified disease events could help distinguish territorial epidemiological variation from inequalities in service access, disease recognition, and reporting capacity.

Taken together, these findings identify priorities for programme evaluation and further investigation rather than causal prescriptions for service delivery.

## 5. Conclusions

This study examined holding-level participation in cattle vaccination and disease-control activities and the reporting of principal cattle disease within the principal continental domain of Ecuador using ESPAC 2025 public-use microdata and survey-design-adjusted analyses. The study distinguished broad regional patterns from a prespecified province-level comparison focused on Manabí.

Reported participation in the official FMD vaccination campaign was substantially more frequent than application of another cattle vaccine or implementation of disease-control activities. Within the principal continental domain, the corresponding estimates were 86.38%, 55.96%, and 64.74%, respectively. These findings indicate the broad holding-level reach of the centrally coordinated FMD programme but do not represent animal-level vaccination coverage, protocol completeness, immunological protection, or the technical quality of the reported activities.

Current herd size was the only farm-level characteristic consistently associated with all four outcomes. Holdings with larger herds had higher adjusted odds of reporting each of the three cattle-health practices and of reporting a principal cattle disease. This pattern may reflect differences in economic exposure, management organisation, veterinary-service contact, the number of animals at risk, observation intensity, and reporting capacity. It does not establish that herd size directly caused either participation in cattle-health activities or disease occurrence.

The adjusted analyses also identified important territorial differences. Holdings in the Amazon and Highlands had lower adjusted odds than holdings in the Coast of reporting another cattle vaccine and disease-control activities, but higher adjusted odds of reporting a principal cattle disease. Holdings in Manabí had higher adjusted odds of reporting all three cattle-health practices than holdings in the other Coast provinces, whereas the Manabí indicator was not associated with self-reported disease. These results identify territorial patterns that warrant further investigation but do not establish causal effects of regional or provincial location.

The estimated proportion of holdings reporting a principal cattle disease was 11.22%. This outcome represents producer recognition and reporting of a heterogeneous group of cattle-health conditions rather than clinical or laboratory-confirmed prevalence. Its associations should therefore be interpreted in relation to disease occurrence, observation, recognition, veterinary access, and reporting capacity. Most of the principal associations remained supported under both singleton-stratum variance specifications, whereas the disease-model associations with no formal education, milk-oriented production, and producer age were variance-sensitive and require particular caution.

The findings provide a holding-level baseline from the final year of routine FMD vaccination and are particularly relevant to Ecuador’s transition towards a surveillance-centred animal-health strategy. Maintaining contact with smaller holdings, producers with limited formal education, and territories with lower reported engagement in cattle-health activities will be important for early recognition, rapid notification, diagnostic investigation, traceability, movement control, biosecurity, and emergency preparedness.

The evidence supports a differentiated veterinary public health strategy in which national animal-health standards are accompanied by territorially adapted service delivery, accessible producer communication, sustained veterinary support, and surveillance systems capable of maintaining contact with diverse cattle-production settings. Repeated analyses of subsequent surveys, pathogen- or syndrome-specific surveillance, qualitative investigation, and more detailed information on veterinary-service access, vaccination protocols, disease-control activities, diagnosis, and notification are needed to evaluate subsequent changes and clarify the mechanisms underlying the observed associations.

## Figures and Tables

**Table 1:** Analytical sample construction and survey-design characteristics.

Characteristic	Principal Continental Domain	Manabí	Santa Elena
**Sample construction**			
Cattle records in the original database	10,715	1423	26
Records with a positive final expansion factor	10,629	1394	26
Positive-weight records assigned to the “undelimited zone” category	41	—	—
Holdings in the principal territorial domain	10,588	1394	26
**Valid observations by outcome**			
Official FMD vaccination campaign	10,588	1394	26
Application of other cattle vaccines	10,588	1394	26
Disease-control activities	10,576	1393	26
Any self-reported principal cattle disease	10,588	1394	26
**Survey-design structure of the territorial domain**			
Sampling strata represented	139	9	4
PSUs represented	3586	404	14
Singleton strata in the complete positive-weight design	58	—	—

Note: Values are unweighted counts. The original cattle-module database contained 10,715 linked records. Design-based estimation was restricted to records with a positive final expansion weight. The principal continental domain was further restricted to positive-weight holdings assigned to one of the 23 provinces of continental Ecuador. Manabí and Santa Elena were analysed as survey domains while retaining the complete survey-design structure for variance estimation. The 58 singleton strata were identified in the complete positive-weight design before exclusion of the “undelimited zone” category. FMD, foot-and-mouth disease; PSU, primary sampling unit.

**Table 2:** Design-adjusted estimates of vaccination, disease-control practices, and self-reported cattle disease.

Outcome	Continental Ecuador, % (95% CI)	Coast, % (95% CI)	Highlands, % (95% CI)	Amazon, % (95% CI)	Manabí, % (95% CI)	Santa Elena, % (95% CI)
Official FMD vaccination campaign	86.38 (85.10–87.66)	92.57 (90.79–94.36)	84.94 (83.29–86.59)	82.92 (78.77–87.07)	95.72 (93.75–97.68)	100.00 (100.00–100.00)
Application of other cattle vaccines	55.96 (53.99–57.92)	79.15 (76.30–82.00)	50.37 (47.93–52.81)	45.82 (39.65–51.99)	85.99 (82.93–89.05)	100.00 (100.00–100.00)
Disease-control activities	64.74 (62.84–66.64)	84.48 (82.10–86.86)	59.60 (57.25–61.94)	61.49 (55.58–67.40)	91.31 (88.74–93.89)	100.00 (100.00–100.00)
Any self-reported principal cattle disease	11.22 (10.25–12.19)	7.91 (6.20–9.63)	11.90 (10.71–13.09)	14.27 (10.62–17.93)	8.11 (5.65–10.56)	14.26 (0.00–48.48)

Note: Estimates incorporate the final expansion factor, sampling strata, and primary sampling units. Coast, Highlands, Amazon, Manabí, and Santa Elena were estimated as survey domains while retaining the complete 23-province survey-design structure for variance estimation. Confidence intervals were calculated using first-order Taylor linearisation and the principal singleton-stratum adjustment. The three estimates equal to 100% for Santa Elena were based on 26 sampled holdings with no observed outcome variation and should not be interpreted as evidence of complete provincial participation. FMD, foot-and-mouth disease; CI, confidence interval.

**Table 3:** Survey-weighted logistic regression model for reported participation in the official FMD vaccination campaign.

Explanatory Variable	aOR	95% CI	*p*-Value
**Formal Education**			
Primary education	Reference	—	—
Higher education	1.49	1.00–2.21	0.051
No formal education	0.52	0.41–0.67	<0.001
Secondary education	0.94	0.72–1.21	0.620
**Cattle-production orientation**			
Dual purpose	Reference	—	—
Meat	1.04	0.81–1.34	0.757
Milk	1.37	1.09–1.74	0.008
Mixed	1.56	0.84–2.91	0.158
Not classified	0.60	0.21–1.74	0.350
**Geographical region**			
Coast	Reference	—	—
Amazon	0.68	0.42–1.10	0.117
Highlands	0.69	0.45–1.05	0.086
Log(1 + current herd size)	2.11	1.82–2.46	<0.001
Log(1 + farm area, ha)	0.70	0.62–0.80	<0.001
Producer age, per 10 years	1.07	1.00–1.15	0.055
Female producer versus male	0.96	0.80–1.14	0.619
Digital-access score, 0–3	0.85	0.77–0.94	0.002
Lives on the holding	0.88	0.72–1.07	0.190
Affiliation with the Rural Social Insurance	0.83	0.67–1.03	0.093
Manabí versus other coastal provinces	2.87	1.57–5.26	<0.001

Note: The model included 10,538 cattle holdings with complete information for the outcome and all covariates. All explanatory variables shown in the table were entered simultaneously. The model incorporated the final ESPAC expansion weight, sampling strata, and PSUs. Variances were estimated using first-order Taylor linearisation and the principal singleton-stratum variance specification. The reference categories were primary education, dual-purpose production, the Coast region, male producer, not living on the holding, and no Seguro Social Campesino affiliation. Because geographical region and the Manabí indicator were included simultaneously, the Manabí coefficient represents the adjusted comparison with holdings in the other Coast provinces. Continuous-variable estimates correspond to the units shown in the table. aOR, adjusted odds ratio; CI, confidence interval; FMD, foot-and-mouth disease; PSU, primary sampling unit.

**Table 4:** Survey-weighted logistic regression models for reported application of another cattle vaccine and implementation of disease-control activities.

Explanatory Variable	Another Cattle Vaccine, aOR (95% CI)	*p*-Value	Disease-Control Activities, aOR (95% CI)	*p*-Value
**Formal education**				
Primary education	Reference	—	Reference	—
Higher education	1.05 (0.82–1.35)	0.701	1.13 (0.85–1.49)	0.406
No formal education	0.83 (0.69–1.00)	0.053	0.76 (0.62–0.92)	0.005
Secondary education	1.07 (0.90–1.27)	0.465	1.07 (0.90–1.29)	0.436
**Cattle-production orientation**				
Dual-purpose	Reference	—	Reference	—
Meat	1.07 (0.90–1.27)	0.461	0.87 (0.72–1.04)	0.114
Milk	1.03 (0.87–1.21)	0.754	0.97 (0.82–1.16)	0.770
Mixed	1.24 (0.77–1.99)	0.378	0.71 (0.47–1.08)	0.108
Not classified	0.85 (0.23–3.08)	0.804	2.28 (0.62–8.36)	0.215
**Geographical region**				
Coast	Reference	—	Reference	—
Amazon	0.34 (0.23–0.50)	<0.001	0.50 (0.33–0.75)	0.001
Highlands	0.46 (0.33–0.64)	<0.001	0.59 (0.42–0.83)	0.002
Log(1 + current herd size)	1.60 (1.44–1.77)	<0.001	1.60 (1.44–1.79)	<0.001
Log(1 + farm area, ha)	0.86 (0.79–0.94)	0.001	0.92 (0.84–1.01)	0.083
Producer age, per 10 years	1.01 (0.96–1.06)	0.740	1.04 (0.98–1.09)	0.193
Female producer	1.04 (0.91–1.19)	0.562	0.98 (0.85–1.14)	0.834
Digital-access score, 0–3	1.05 (0.97–1.13)	0.233	1.05 (0.97–1.13)	0.246
Lives on the holding	0.99 (0.85–1.14)	0.845	1.09 (0.94–1.27)	0.234
Affiliation with the Rural Social Insurance Scheme	1.02 (0.86–1.19)	0.850	1.03 (0.87–1.23)	0.695
Manabí versus other Coast provinces	2.33 (1.57–3.45)	<0.001	2.90 (1.86–4.51)	<0.001

Note: The model for reported application of another cattle vaccine included 10,538 holdings with complete outcome and covariate information. The disease-control-activities model included 10,526 holdings because of outcome-specific missing information. All explanatory variables shown were entered simultaneously in both models. The models incorporated the final ESPAC expansion weight, sampling strata, and PSUs. Variances were estimated using first-order Taylor linearisation and the principal singleton-stratum variance specification. The reference categories were primary education, dual-purpose production, the Coast region, male producer, not living on the holding, and no affiliation with the Rural Social Insurance Scheme. Because geographical region and the Manabí indicator were included simultaneously, the Manabí coefficient represents the adjusted comparison with holdings in the other Coast provinces. Continuous-variable estimates correspond to the units shown in the table. aOR, adjusted odds ratio; CI, confidence interval; FMD, foot-and-mouth disease; PSU, primary sampling unit.

**Table 5:** Survey-weighted logistic regression model for any self-reported principal cattle disease.

Explanatory Variable	aOR	95% CI	*p*-Value
**Formal education**			
Primary education	Reference	—	Reference
Higher education	0.97	0.67–1.39	0.849
No formal education †	1.36	1.04–1.76	0.022
Secondary education	0.90	0.70–1.15	0.410
**Cattle-production orientation**			
Dual-purpose	Reference	—	Reference
Meat	0.60	0.47–0.77	<0.001
Milk †	1.31	1.06–1.61	0.011
Mixed	0.94	0.51–1.75	0.848
Not classified	1.47	0.34–6.44	0.607
**Geographical region**			
Coast	Reference	—	Reference
Amazon	2.31	1.47–3.63	<0.001
Highlands	2.70	1.86–3.91	<0.001
Log(1 + current herd size)	1.81	1.58–2.09	<0.001
Log(1 + farm area, ha)	1.06	0.94–1.18	0.359
Producer age, per 10 years †	0.93	0.87–0.99	0.027
Female producer	1.04	0.86–1.25	0.712
Digital-access score, 0–3	1.13	1.04–1.24	0.005
Lives on the holding	1.27	1.07–1.51	0.006
Affiliation with the Rural Social Insurance Scheme	1.39	1.12–1.73	0.003
Manabí versus other Coast provinces	0.85	0.52–1.37	0.493

Note: The model included 10,538 cattle holdings with complete outcome and covariate information. The outcome was coded as 1 when the respondent identified milk fever, mastitis, tympanism, brucellosis, vesicular disease, or another condition as the principal cattle disease and as 0 when no principal disease was reported. The outcome therefore represents producer-reported disease occurrence rather than a clinical, laboratory-confirmed, or administratively verified diagnosis. All explanatory variables shown were entered simultaneously. The model incorporated the final ESPAC expansion weight, sampling strata, and PSUs. Variances were estimated using first-order Taylor linearisation and the principal singleton-stratum variance specification. The reference categories were primary education, dual-purpose production, the Coast region, male producer, not living on the holding, and no affiliation with the Rural Social Insurance Scheme. Because geographical region and the Manabí indicator were included simultaneously, the Manabí coefficient represents the adjusted comparison with holdings in the other Coast provinces. Continuous-variable estimates correspond to the units shown in the table. † Association statistically supported under the principal variance specification but not under the alternative singleton-stratum specification. aOR, adjusted odds ratio; CI, confidence interval; PSU, primary sampling unit.

## Data Availability

The original ESPAC 2025 public-use databases and their official methodological documentation are publicly available from Ecuador’s Instituto Nacional de Estadística y Censos through the official ESPAC database portal [17]. The privacy-reduced analytical dataset, data dictionary, variable-selection and derivation rules, data-processing scripts, survey-analysis code, data-quality audit outputs, design-adjusted estimates, complete regression results, singleton-stratum sensitivity analyses, collinearity diagnostics, and figure source data supporting this study are openly available in Zenodo at https://zenodo.org/records/21621133 [27]. Core reproducibility materials are also provided in Supplementary File S1. The original INEC public-use databases are not redistributed in either the Zenodo record or the Supplementary Material. The analyses, interpretations, and conclusions presented in this study are the responsibility of the authors.

## Supplementary Materials

The following supporting information can be downloaded at: https://www.mdpi.com/article/10.3390/vetsci13080819/s1. Supplementary File S1. ESPAC 2025 Cattle Health Data and Code v1.0.0.

## Abbreviations

The following abbreviations are used in this manuscript:

AGROCALIDAD	Agencia de Regulación y Control Fito y Zoosanitario (Agency for Phytosanitary and Zoosanitary Regulation and Control)
aOR	adjusted odds ratio
CI	confidence interval
ESPAC	Continuous Agricultural Production and Area Survey (*Encuesta de Superficie y Producción Agropecuaria Continua*)
FMD	foot-and-mouth disease
INEC	National Institute of Statistics and Censuses (Instituto Nacional de Estadística y Censos)
PSU	primary sampling unit
WOAH	World Organisation for Animal Health
