# Supplementary material for: Territorial and Farm-Level Inequalities in Cattle Vaccination and Disease-Control Practices in Ecuador: Evidence from the 2025 National Agricultural Survey
Source: Vet Sci. 2026 Aug 17;13(8):819. doi: 10.3390/vetsci13080819 (PMC13517923; doi:10.3390/vetsci13080819)
Supplement: Supplementary file 1 [file vetsci-13-00819-s001.zip › File S1. ESPAC 2025 Cattle Health Data and Code v1.0.0/Figures/Main_Manuscript/Figure_2_Design_Adjusted_Holding_Level_Estimates.pdf]

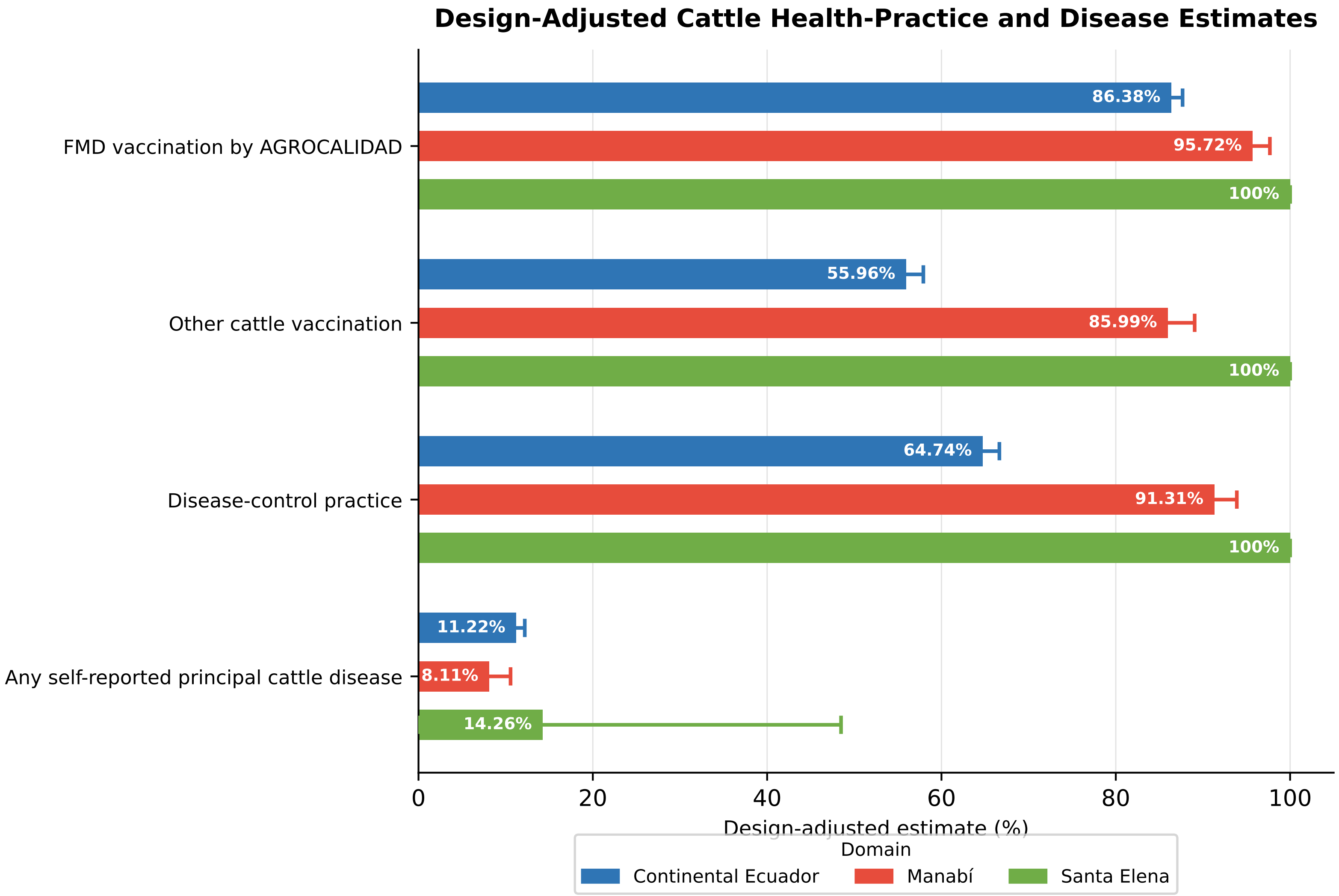

Design-Adjusted Cattle Health-Practice and Disease Estimates
FMD vaccination by AGROCALIDAD
Other cattle vaccination
Disease-control practice
Any self-reported principal cattle disease
86.38%
95.72%
100%
55.96%
85.99%
100%
64.74%
91.31%
100%
11.22%
8.11%
14.26%
0
20
40
60
80
100
Design-adjusted estimate (%)
Domain
Continental Ecuador
Manabí
Santa Elena
