# Supplementary material for: Territorial and Farm-Level Inequalities in Cattle Vaccination and Disease-Control Practices in Ecuador: Evidence from the 2025 National Agricultural Survey
Source: Vet Sci. 2026 Aug 17;13(8):819. doi: 10.3390/vetsci13080819 (PMC13517923; doi:10.3390/vetsci13080819)
Supplement: Supplementary file 1 [file vetsci-13-00819-s001.zip › File S1. ESPAC 2025 Cattle Health Data and Code v1.0.0/Figures/Supplementary/Supplementary_Figure_S1.pdf]

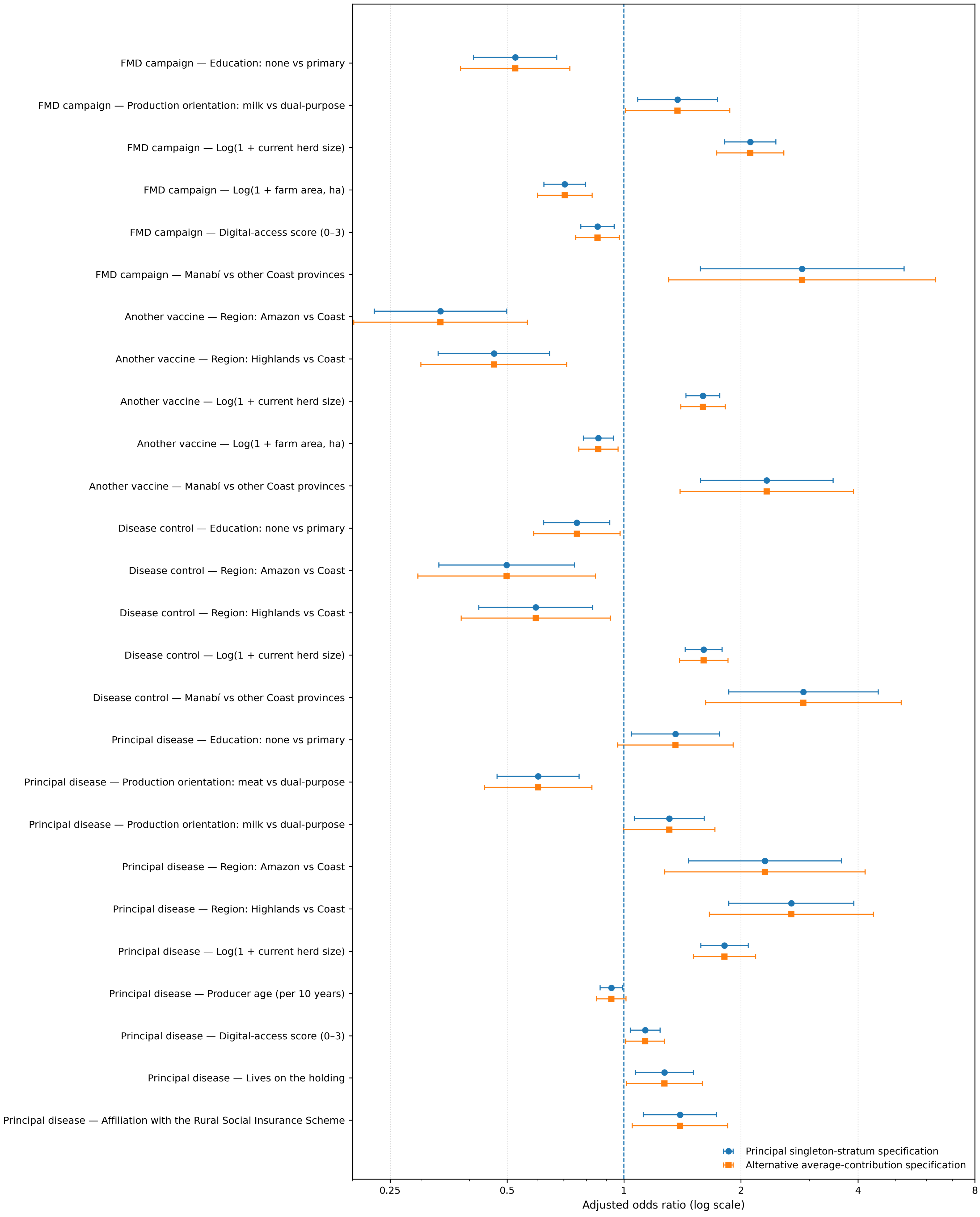

Figure S1. Comparison of adjusted associations under the principal and alternative singleton-stratum variance specifications.

Note: Points represent adjusted odds ratios, and horizontal lines represent 95% confidence intervals. Point estimates are identical under both specifications because the sensitivity analysis modified variance estimation rather than coefficient estimation. The figure includes coefficients statistically supported under the principal variance specification. The vertical dashed line indicates an adjusted odds ratio of 1. FMD, foot-and-mouth disease.
